# Antioxidants reveal an inverted U‐shaped dose‐response relationship between reactive oxygen species levels and the rate of aging in *Caenorhabditis elegans*

**DOI:** 10.1111/acel.12528

**Published:** 2016-09-28

**Authors:** David Desjardins, Briseida Cacho‐Valadez, Ju‐Ling Liu, Ying Wang, Callista Yee, Kristine Bernard, Arman Khaki, Lionel Breton, Siegfried Hekimi

**Affiliations:** ^1^ Department of BiologyMcGill UniversityMontrealQCCanadaH3A 1B1; ^2^ L'Oréal Research and InnovationAulnay sous bois93600France

**Keywords:** aging, antioxidants, *C. elegans*, prooxidants, reactive oxygen species

## Abstract

Reactive oxygen species (ROS) are potentially toxic, but they are also signaling molecules that modulate aging. Recent observations that ROS can promote longevity have to be reconciled with the numerous claims about the benefits of antioxidants on lifespan. Here, three antioxidants [*N*‐acetylcysteine (NAC), vitamin C, and resveratrol (RSV)] were tested on *Caenorhabditis elegans* mutants that alter drug uptake, mitochondrial function, and ROS metabolism. We observed that like pro‐oxidants, antioxidants can both lengthen and shorten lifespan, dependent on concentration, genotypes, and conditions. The effects of antioxidants thus reveal an inverted U‐shaped dose–response relationship between ROS levels and lifespan. In addition, we observed that RSV can act additively to both NAC and paraquat, to dramatically increase lifespan. This suggests that the effect of compounds that modulate ROS levels can be additive when their loci of action or mechanisms of action are sufficiently distinct.

## Introduction

Reactive oxygen species (ROS), such as superoxide and peroxide, are potentially toxic by‐products of energy metabolism as well as signaling molecules (Holmstrom & Finkel, 2014). Both types of properties have been firmly implicated in the aging process, in particular in the nematode *Caenorhabditis elegans* (Van Raamsdonk & Hekimi, 2010; Yang & Hekimi, 2010a). One of the obstacles for understanding the role of ROS in any process is the difficulty of visualizing them directly *in vivo*, as they occur at low concentration, interact readily with other molecules, and are removed by powerful enzymatic systems. An indirect method to overcome this difficulty is to measure ROS damage to macromolecules. However, simply measuring ROS damage cannot reveal any causal role ROS might play in the aging process. Because of this, many studies have relied on studying the effects of exogenous compounds that increase the generation of ROS (pro‐oxidants) or compounds that help to neutralize ROS (antioxidants).

An alternative approach consists in studying mutations that simultaneously alter mitochondrial function, ROS metabolism, and lifespan. Such mutations have been identified in *C. elegans*. They include mutations in *clk‐1, isp‐1*,* nuo‐6*, and *sod‐2* which are long‐lived, as well as *gas‐1*, which was originally identified as a short‐lived mutant. CLK‐1 is necessary for the biosynthesis of ubiquinone (coenzyme Q), the obligate diffusible electron carrier in the mitochondrial electron transport chain (Ewbank *et al*., 1997), and *clk‐1* mutants might have high mitochondrial ROS (mtROS) but low cytoplasmic ROS (Shibata *et al*., 2003; Schaar *et al*., 2015). ISP‐1 is the ‘Rieske’ iron sulfur protein of mitochondrial complex III (Feng *et al*., 2001). NUO‐6 and GAS‐1 are subunits of mitochondrial complex I (Kayser *et al*., 2001; Yang & Hekimi, 2010b). Mutations in all these genes result in defective mitochondrial function and increased mtROS generation (Van Raamsdonk & Hekimi, 2010; Yang & Hekimi, 2010a). In fact, the longevity of *isp‐1* and *nuo‐6* mutants depends on an elevated generation of mitochondrial superoxide, which triggers the apoptotic signaling pathway without inducing apoptosis (Yee *et al*., 2014). Strikingly, this effect of the mutations can be phenocopied by treatment with low levels (~0.1 ± 0.05 mm) of the mitochondrial superoxide generator paraquat (PQ; Yang & Hekimi, 2010a).

Superoxide dismutases (SODs) are highly conserved enzymes that convert superoxide into hydrogen peroxide. The almost universal presence of SOD enzymes in organisms strongly suggests that superoxide detoxification is necessary for cellular life. However, recent findings suggest that superoxide detoxification by SOD enzymes is not involved in the aging process. Indeed, knocking out all five *C. elegans* SODs in the same strain (abbreviated *sod‐12345*) does not shorten lifespan despite elevated oxidative stress (Yang & Hekimi, 2010a; Van Raamsdonk & Hekimi, 2012).

The banned toxic herbicide PQ (1,1′‐dimethyl‐4,4′‐bipyridinium dichloride) is a pro‐oxidant that increases superoxide generation, in particular in mitochondria (Cocheme & Murphy, 2008), but possibly in other locations as well (Labuschagne *et al*., 2013). Treatment of wild‐type worms (N2) with PQ results in an inverted U‐shaped dose–response relationship with a maximal lifespan‐lengthening effect at 0.1 mm (Yang & Hekimi, 2010a; Van Raamsdonk & Hekimi, 2012). The placement of the dose–response relationship along the two axes (lifespan and PQ concentration) depends on the genotype tested (Hwang *et al*., 2014). These observations suggest that there is also an inverted U‐shaped relationship between mtROS levels and lifespan (Van Raamsdonk & Hekimi, 2012). This model potentially explains various observations. For example, treatment of *isp‐1(qm150)* and *nuo‐6(qm200)* with antioxidants leads to a shortening of their extended lifespans (Yang & Hekimi, 2010a; Van Raamsdonk & Hekimi, 2012).

Vitamin C (VitC) is chemically an antioxidant and is used as such in disease prevention (Padayatty *et al*., 2003). Although addition of VitC to the growth medium of *C. elegans* does not much affect the wild‐type, it does so when included in liposomes (Shibamura *et al*., 2009).

*N*‐acetylcysteine (NAC) is a modified version of the amino acid cysteine with the acetyl group attached to its nitrogen atom aiding in its transport across membranes. Cysteine can be released from NAC by hydrolysis within cells (Rushworth & Megson, 2014). The cysteine provides an amino acid source for the synthesis of glutathione. In *C. elegans*, NAC has been shown to suppress increased ROS generation (Schulz *et al*., 2007; Yang & Hekimi, 2010a). NAC treatment does not increase the lifespan of the wild‐type when simply added to the growth medium and is toxic above 10 mm (Yang & Hekimi, 2010a). However, it is capable of having beneficial effects when it is administered in liposomes (Shibamura *et al*., 2009).

Resveratrol (RSV) is a natural plant product that has been very widely studied for its potential beneficial effects on the redox balance and is used as antiaging dietary supplement. This is despite the fact that there is no consensus as to its modes of action (Bhullar & Hubbard, 2015; Bitterman & Chung, 2015). In particular, RSV might act as an antioxidant, but other redox effects have also been documented (e.g., Miura *et al*., 2000; Stivala *et al*., 2001; Galati *et al*., 2002; Ahmad *et al*., 2003, 2005; Zheng *et al*., 2006; de la Lastra & Villegas, 2007; Gadacha *et al*., 2009; Queiroz *et al*., 2009; Gueguen *et al*., 2015). In addition, numerous distinct specific targets have been identified and characterized (reviewed in Bhullar & Hubbard, 2015; Bitterman & Chung, 2015). The effect of RSV on lifespan per se has also been widely tested and positive effects documented in both invertebrates and vertebrates (Bhullar & Hubbard, 2015). In *C. elegans*, a relatively small but reproducible lifespan‐extending effect has been documented by several groups (Wood *et al*., 2004; Viswanathan *et al*., 2005; Bass *et al*., 2007; Agarwal & Baur, 2011; Morselli *et al*., 2010; Zarse *et al*., 2010).

Here, we show (i) that lifespan changes produced by antioxidant treatment of *C. elegans* can yield inverted U‐shaped dose–response relationships, (ii) that the degree of penetration of compounds determines the outcome of antioxidant action, (iii) that altered ROS generation or detoxification can determine the outcome of antioxidant action, and (iv) that RSV can act additively to both the antioxidant NAC and the pro‐oxidant PQ. Together with previous data from pro‐oxidant treatment, these observations strongly suggest the existence of an inverted U‐shaped relationship between ROS levels and lifespan. We also propose a model in which the effect on lifespan of any mutation or compound that alters ROS levels is a combination of the beneficial and deleterious effects of ROS in all the cell types and subcellular sites that are affected by the mutation or reached by the compound.

## Results

### NAC, VitC, RSV, and PQ extend wild‐type lifespan

**Figure 1:**
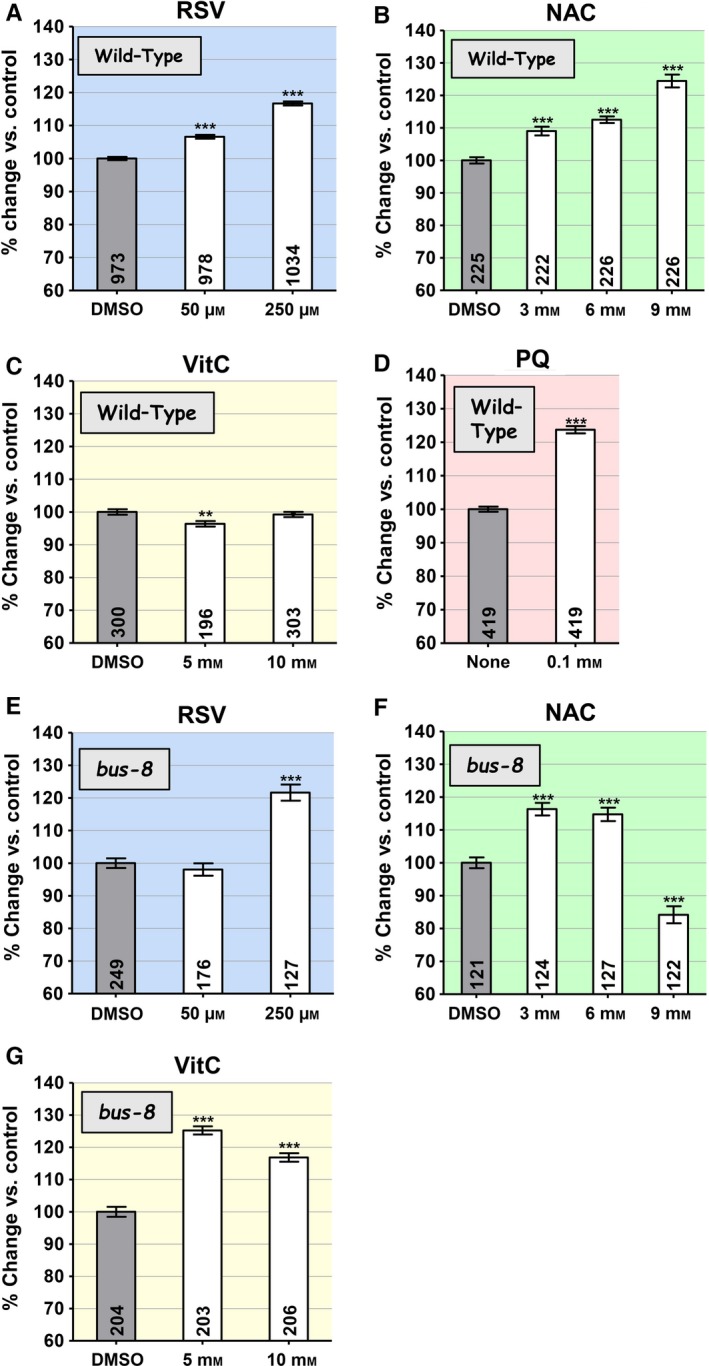
The effect of the antioxidants resveratrol (RSV), *N*‐acetylcysteine (NAC), and vitamin C (VitC) as well as of the pro‐oxidant paraquat (PQ), on the lifespan of wild‐type worms and *bus‐8* mutants, which have increased cuticle permeability. The panels are color‐coded by compound. The mean lifespans are given as percent change in average lifespan vs. untreated control. The *y*‐axis of all graphs shows changes over a 60–140% range. Sample sizes are given in the bars. Error bars are SEM. The asterisks indicate *P*‐values from *t*‐test comparisons to the control: **P <* *0.01 and ***P <* *0.0001. All compounds except VitC increase lifespan at one or several concentrations (A, B, D). VitC only very slightly shortens lifespan at the lowest concentration of VitC (C). Increased penetration due to the *bus‐8* mutation abolishes the effect of RSV at 50 μm, allows VitC to have a strong effect at both concentration, and reveals an inverted U‐shaped dose–response relationship with lifespan for NAC and VitC.

Resveratrol is relatively insoluble in aqueous media and needs to be dissolved in DMSO for addition to standard nematode growth medium (NGM) plates. To allow for appropriate comparisons, we tested all three antioxidants (RSV, NAC, and VitC) at the same concentration of DMSO, although NAC and VitC are readily soluble in NGM without DMSO. The maximum soluble concentration of RSV in DMSO was 250 μm. The range of concentrations for NAC and VitC was chosen based on previous work. PQ however was simply dissolved in water and was only tested at one concentration (0.1 mm), as the inverted U‐shaped dose dependency of its lifespan‐lengthening effects is already well documented (Van Raamsdonk & Hekimi, 2012). Under conditions used, NAC, RSV, and PQ were capable of increasing wild‐type lifespan in a dose‐dependent manner (Fig. 1A–D). VitC however produced only a very mild lifespan shortening at the lower concentration tested (5 mm). All numerical data from which the graphs in all figures were drawn are given in Table S1 (Supporting information). We had previously found that NAC was without effect on wild‐type lifespan when provided without DMSO (Yang & Hekimi, 2010a). We therefore directly tested the effect of DMSO on lifespan in the presence of NAC and found that its presence indeed allowed for lifespan lengthening (Table S1).

### Effects of altered compound uptake

Mutations in the gene *bus‐8* alter the worm's cuticle in such a way as to increase its permeability to exogenous compounds (Partridge *et al*., 2008). The *bus‐8(e2698)* mutants show a wild‐type lifespan in the absence of any treatment (Table S1). We found however that treatment of *bus‐8* mutants with antioxidants resulted in outcomes on lifespan that were qualitatively different from those of treatment of the wild‐type. Treatment with RSV at the lower concentration of 50 μm (Fig. 1E) produced no effect, in contrast to what was observed for the wild‐type (Fig. 1A), while the effect of treatment at 250 μm was indistinguishable from that of treatment of the wild‐type. More dramatically, treatment with NAC had the largest effect at the lower concentrations of 3 and 6 mm and shortened lifespan at the highest concentration (9 mm; Fig. 1F). Finally, the most striking difference to the wild‐type was observed with VitC (Fig. 1G). *bus‐8* mutants showed a particularly large increase in lifespan at the lower concentration of 5 mm (which slightly shortens wild‐type lifespan) and a lesser, albeit still large, effect at 10 mm (which is without effect on the wild‐type). It is unknown whether the *bus‐8* mutation simply increases the amount of compound entering the organism or whether it allows for access to additional tissues, or both.

### Effects of NAC on mutants with altered mitochondrial function

**Figure 2:**
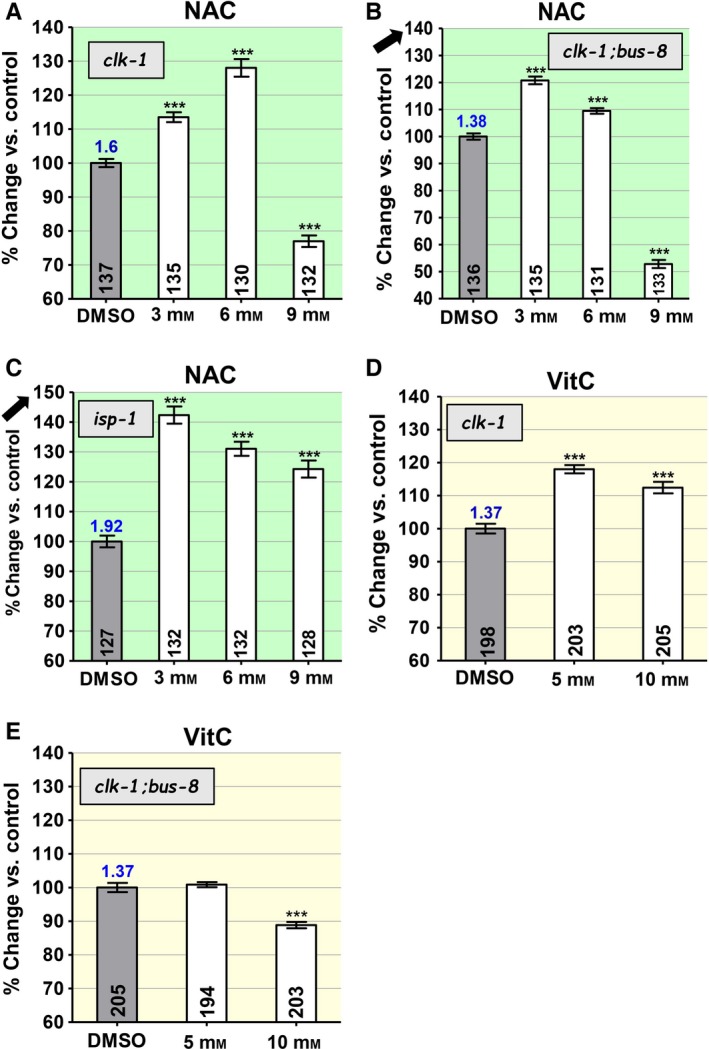
The effects of the antioxidant *N*‐acetylcysteine (NAC) and vitamin C (VitC) on the lifespans of mutants with altered mitochondrial function and Reactive oxygen species (ROS) generation (*clk‐1* and *isp‐1*) and double mutants that also include a *bus‐8* mutation, which increases cuticle permeability (*clk‐1;bus‐8* and *isp‐1;bus‐8*). The panels are color‐coded by compound. The mean lifespans are given as percent change in average lifespan vs. untreated control. The *y*‐axis of most graphs shows changes over a 60–140% range (A, D, E). The arrows pointing to numbers on the *y*‐axis in B and C indicate that a different range is shown, down to 40% and up to 150%, respectively. The ratio of the lifespan of untreated worms of the genotype represented in the graph to the lifespan of wild‐type worms treated only with DMSO is given as a number on top of the bar corresponding to untreated worms of the experimental genotype (see Table S1 for all values). Sample sizes are given in the bars. Error bars are SEM. The asterisks indicate *P*‐values from *t*‐test comparisons to the control: ***P <* *0.0001. The effect of NAC on the lifespan of *clk‐1* and *isp‐1* reveals an inverted U‐shaped dose–response relationship (A, C), as does the effect of VitC on *clk‐1* (D). The effects of the two concentrations of VitC are in fact different from each other (*P *<* *0.01). Increasing cuticle permeability in double mutants that include *bus‐8* in *clk‐1;bus‐8* double mutants reinforces the effect of NAC and VitC, equivalent to a shift to the left of the dose–response relationship.

Loss of CLK‐1 function leads to diminished mitochondrial function, elevated mitochondrial oxidative stress, and a longer lifespan in *clk‐1(qm30)* and *clk‐1(e2519)* mutants (Felkai *et al*., 1999; Shibata *et al*., 2003). The effects of NAC treatment on *clk‐1(qm30)* (Fig. 2A) are similar to those of treatment of *bus‐8* (Fig. 1F). Although NAC lengthens lifespan robustly at the lower concentrations, it severely shortens lifespan at the highest concentration (9 mm). The effects of the two mutations seem to be partially additive in *clk‐1;bus‐8* double mutants, which show a greater decline in lifespan lengthening with increasing concentration and a very severe lifespan shortening at the highest concentration (Fig. 2B). This suggests that *bus‐8* indeed increases the concentration of NAC at the target that leads to shortened lifespan of *clk‐1(qm30)* at higher concentrations of NAC (Fig. 2A).

The *isp‐1(qm150)* point mutation slows mitochondrial electron transport and increases mitochondrial superoxide generation but not general oxidative stress or sensitivity to loss of superoxide detoxification (Feng *et al*., 2001; Yang *et al*., 2007; Yang & Hekimi, 2010a). Treatment of *isp‐1(qm150)* with NAC at the lowest concentration leads to a particularly dramatic increase in lifespan of these already very long‐lived mutants (Fig. 2C). However, this positive effect on lifespan gradually diminishes at higher concentrations, thus revealing an inverted U‐shaped dose–response relationship as for *bus‐8* and *clk‐1*, albeit not as marked. In a previous study, NAC treatment in the absence of DMSO was found to decrease rather than increase the lifespan of *isp‐1(qm150)* (Yang & Hekimi, 2010a). Thus, it appears that the presence of DMSO allows NAC to reach targets not reached without DMSO, and where its effects are lifespan‐lengthening, with the inverted U‐shaped relationship observed in Fig. 2C thus resulting from a combination of lifespan‐lengthening and lifespan‐shortening effects on different targets.

### Effects of VitC on mutants with altered mitochondrial function

Although VitC has almost no effects on the wild‐type (Fig. 1C), it is lifespan‐lengthening on *clk‐1(qm30)* mutants at both concentrations, but the effect is greatest at the lower concentration of 5 mm (Fig. 2D). Furthermore, in *clk‐1;bus‐8* double mutants there is no beneficial effect at 5 mm and a lifespan‐shortening effect at 10 mm (Fig. 2E). This suggest that VitC is lifespan‐shortening when its concentration and penetration are high, with the absence of effect of 5 mm in *clk‐1;bus‐8*, the result of a balance between lifespan‐lengthening and lifespan‐shortening effects. The effect of VitC on *isp‐1* has been previously shown to be lifespan‐shortening, which was not retested in the present study (Van Raamsdonk & Hekimi, 2012).

### Effects of RSV on mutants with altered mitochondrial function

**Figure 3:**
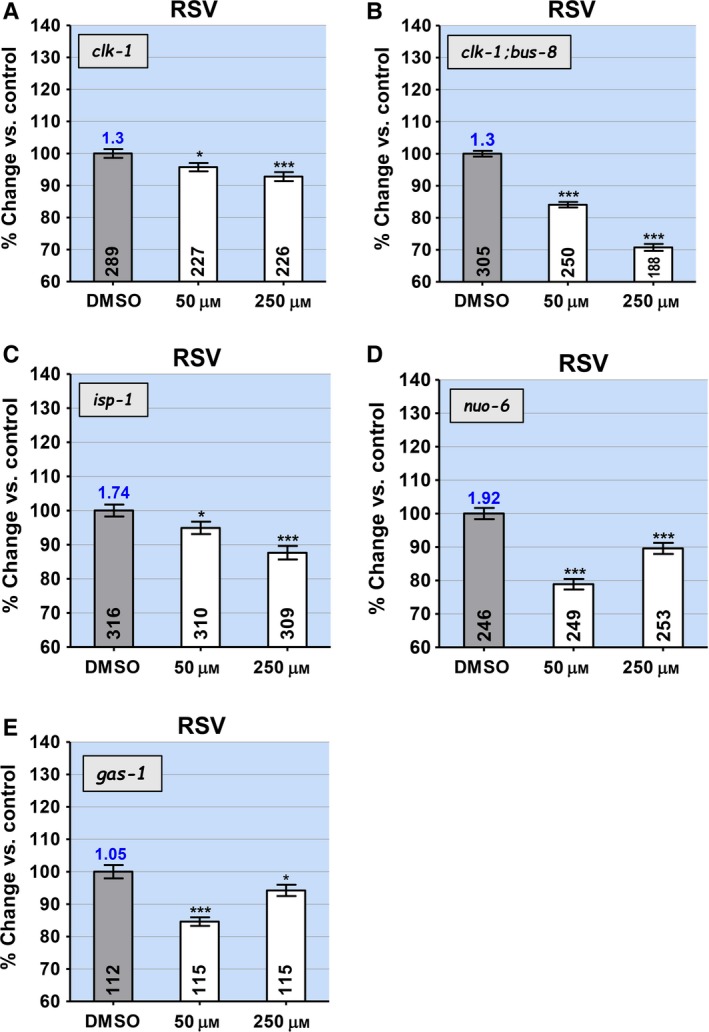
The effect of resveratrol (RSV) on the lifespan of mutants with altered mitochondrial function and reactive oxygen species (ROS) generation (*clk‐1, isp‐1, nuo‐6*, and *gas‐1*) and on *clk‐1;bus‐8* double mutants, which have increased cuticle permeability. The ratio of the lifespan of untreated worms of the genotype represented in the graph to the lifespan of wild‐type worms treated only with DMSO is given as a number on top of the bar corresponding to untreated worms of the experimental genotype (see Table S1 for all values). Sample sizes are given in the bars. Error bars are SEM. The asterisks indicate *P*‐values from *t*‐test comparisons to the control: *P <* *0.05 and ***P <* *0.0001. The effect of RSV is lifespan‐shortening for *clk‐1* and *isp‐1* mutants (A, C), and the presence of *bus‐8* severely enhances this effect in *clk‐1:bus‐8* mutants (B). The effect of RSV on *nuo‐6* and *gas‐1* mutants reveals a U‐shaped, rather than inverted U‐shaped, dose–response relationship (D, E).

Although RSV lengthens the lifespan of the wild‐type (Fig. 1A), we found that at all concentrations, it shortens the lifespan of *clk‐1(qm30)* (Fig. 3A), *clk‐1(e2519)* (Table S1) and *isp‐1(qm150)* (Fig. 3C). As before, increased permeability in *clk‐1;bus‐8* resulted in much more severe effects (Fig. 3B). To explore further the properties of RSV, we tested it on two additional mutants with defects in subunits of respiratory chain complexes that result in altered lifespan. *nuo‐6(qm200)* is a mutant of a subunit of complex I with a phenotype similar to *isp‐1* (Yang & Hekimi, 2010b). *gas‐1(fc21)* is a mutant in a subunit of complex I with a short lifespan that is rescued and even lengthened by treatment with fluorodeoxyuridine (FUDR) by an unknown mechanism (Kayser *et al*., 2001; Van Raamsdonk & Hekimi, 2011). Treatment of *nuo‐6* and *gas‐1* with RSV shortened lifespan at both concentrations tested, but with a much more pronounced effect at the lower concentration (Fig. 3D,E). Thus, the dose–response relationship here appears to be U‐shaped rather than inverted U‐shaped as observed for the effects of NAC and VitC on various genotypes.

### The lifespan of *gas‐1* is lengthened by PQ

**Figure 4:**
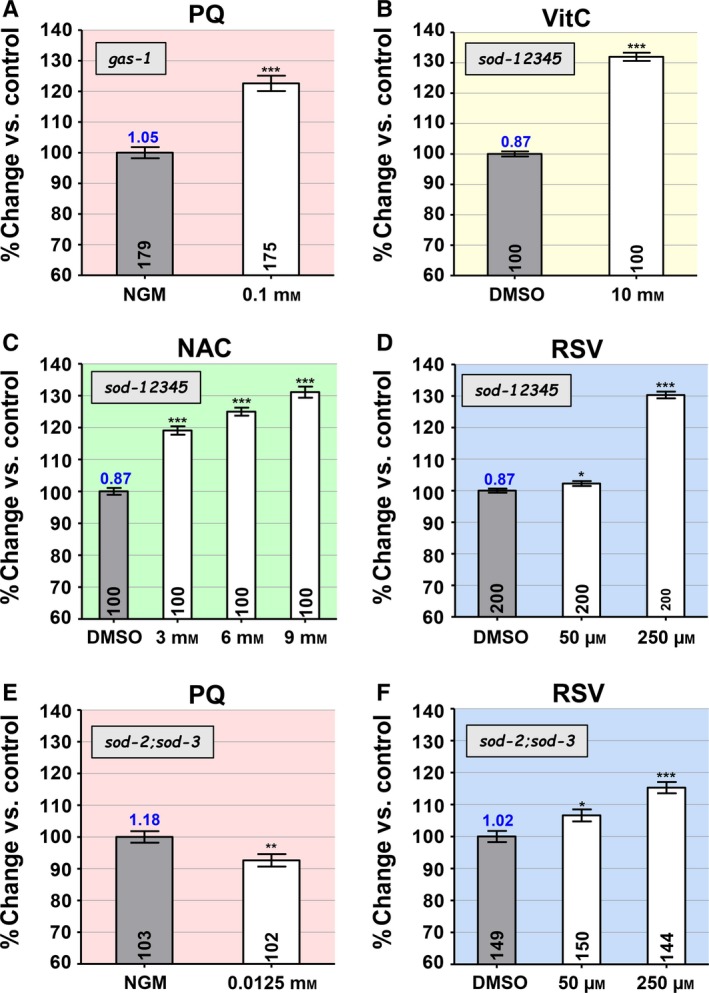
The effect of the pro‐oxidant paraquat (PQ) and the antioxidants resveratrol (RSV), vitamin C (VitC), and *N*‐acetylcysteine (NAC) on the lifespans of the complex I mutants *gas‐1*, of mutants simultaneously missing all five superoxide dismutases (SODs) (abbreviated *sod‐12345*), and of mutants missing only the two mitochondrial SODs (*sod‐2;sod‐3* double mutants). The mean lifespans are given as percent change in average lifespan vs. untreated control. The *y*‐axis of all bar graphs shows changes over a 60–140% range. The ratio of the lifespan of untreated worms of the genotype represented in the graph to the lifespan of wild‐type worms treated only with DMSO is given as a number on top of the bar corresponding to untreated worms of the experimental genotype (see Table S1 for all values). Sample sizes are given in the bars. Error bars are SEM. The asterisks indicate *P*‐values from *t*‐test comparisons to the controls. For all the bar graphs, *P <* *0.05, **P <* *0.01, and ***P <* *0.0001. PQ increases the lifespan *gas‐1* (A) despite the fact that these mutants sustain elevated mitochondrial oxidative stress. All three antioxidants strongly increase the survival of sod*‐12345* mutants (C–E), suggesting that RSV acts like an antioxidant in *C. elegans*. A dosage of PQ (0.0125 mm) that is eight times lower than the dosage for optimal lifespan lengthening of the wild‐type (0.1 mm) still significantly shortens the lifespan of *sod‐2;sod‐3* double mutants (E), suggesting that these mutants sustain severe mitochondrial oxidative stress. However, the effect of RSV on these mutants is indistinguishable from its effect on the wild‐type (F), thus suggesting that RSV does not act on mitochondria.

To explore the significance of the similarity of reaction of *nuo‐6* and *gas‐1* to RSV, we tested the effect of PQ on *gas‐1*, which had not previously been determined. The treatment produced a robust 26% and 23% increase in mean and maximum lifespan, respectively (Fig. 4A and Table S1), similar to that observed with the wild‐type. Thus, it appears that an increase in ROS induced by PQ in at least some cell types can be beneficial in a mutant such as *gas‐1*, which sustains mitochondrial dysfunction and elevated mitochondrial oxidative stress, and which is not long‐lived. Possibly, the particular defect in *gas‐1* does not increase mtROS in the most relevant cell type, which can however be induced there by PQ.

### Effect of RSV on mutants entirely lacking superoxide dismutase activity

We have previously studied a mutant strain missing all five SODs (abbreviated *sod‐12345*) (Van Raamsdonk & Hekimi, 2012). The wild‐type lifespan of this strain is believed to be the result of a balance between the deleterious effect of toxic high ROS levels and their pro‐longevity effects. As expected, no concentration of PQ is capable of increasing this strain's lifespan; rather, PQ decreases its lifespan even at a very low concentration (above 0.01 mm). We also previously showed that treatment with VitC at a concentration that had virtually no effect on the wild‐type could robustly extend the quintuple mutant's lifespan (Van Raamsdonk & Hekimi, 2012). We have now confirmed this effect of VitC (Fig. 4B) and found that NAC also strongly increased the strain's lifespan at all tested concentrations (Fig. 4C). Furthermore, we found that RSV at 250 μm increases the lifespan of the strain as strongly as VitC and NAC (Fig. 4D), indicating that RSV indeed acts as an antioxidant on the lifespan of *C. elegans*.

### Effects of RSV and PQ on *sod‐2;sod‐3* double mutants

To attempt to narrow down the site of action of RSV, we tested its action on mutants lacking both mitochondrial SODs, SOD‐2 and SOD‐3. As expected, these mutants are hypersensitive to lifespan‐shortening treatments with various pro‐oxidants (Van Raamsdonk & Hekimi, 2009). Here, we tested an eightfold lower dosage (0.0125 mm) of the mitochondrial pro‐oxidant PQ than that is required for its maximum pro‐longevity effect, and this still resulted in significant lifespan shortening for these mutants (Fig. 4E), indicating that they sustain severe mitochondrial oxidative stress. Yet, the lifespan‐lengthening effect of RSV on these mutants (Fig. 4F) is neither lessened nor enhanced compared to the effect on the wild‐type (Fig. 1A). This suggests that the mechanism of action of RSV does not implicate mitochondria.

### Additive effects of RSV and NAC

**Figure 5:**
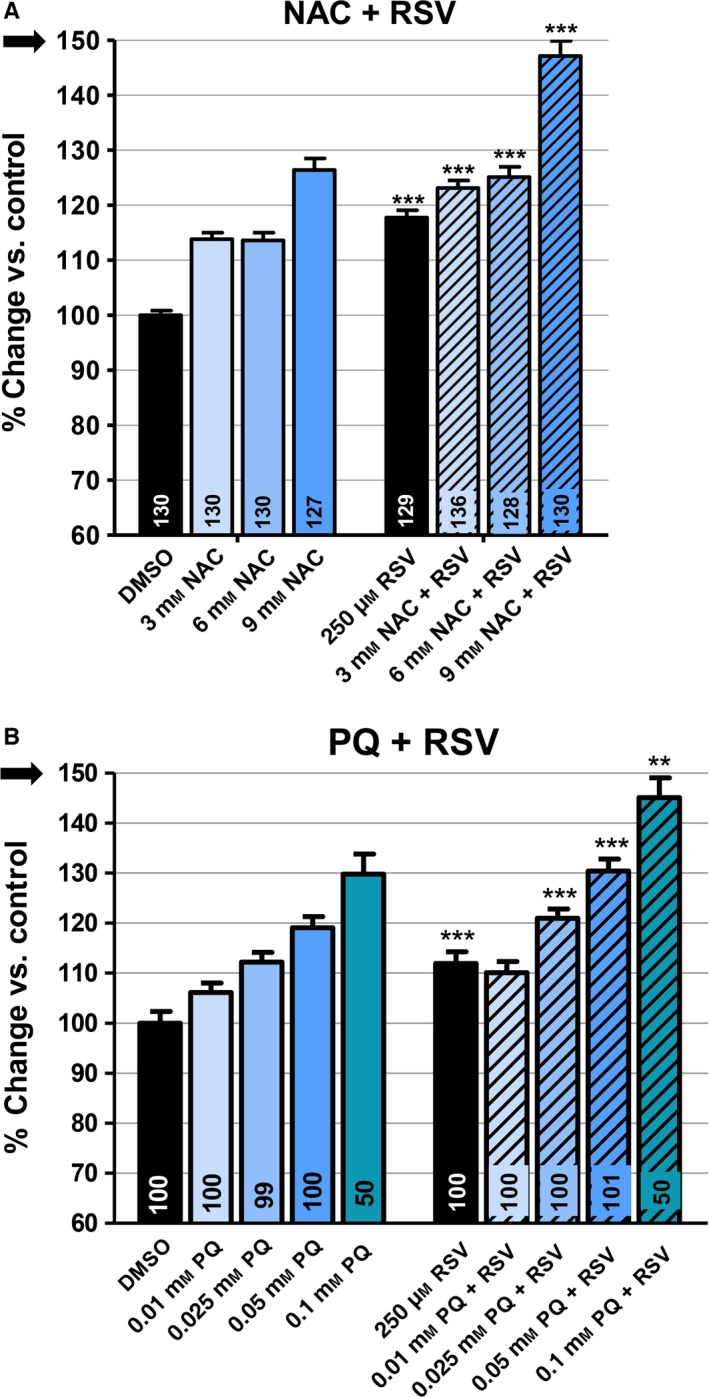
The effects on lifespan of simultaneously treating wild‐type worms with resveratrol (RSV) and either *N*‐acetylcysteine (NAC) or paraquat (PQ). The mean lifespans are given as percent change in average lifespan vs. untreated control (DMSO only). The *y*‐axis of all bar graphs shows changes over a 60–150% range. Arrows indicate that the *y*‐axes show a greater range than in most other graphs in the study. Sample sizes are given in the bars. Error bars are SEM. The asterisks indicate *P*‐values from *t*‐test comparisons of the value for worms treated with RSV and NAC, or RSV and PQ, to the value of the equivalent treatment of only NAC or only PQ (the left set of bars): ***P <* *0.0001. The effect of RSV appears to be fully additive to the effects of NAC(A) and PQ(B).

The activity on lifespan of all three antioxidants tested is altered by mutations that affect mitochondrial function, ROS generation, and cuticle permeability. However, the effects of the mutations are different for each antioxidant as illustrated by the following two examples: (i) NAC lengthens, but RSV shortens, the lifespan of *clk‐1* and *isp‐1*; and (ii) the increased permeability of *bus‐8* mutants hugely increases the pro‐longevity effect of VitC, yet also weakens the effect of RSV at 50 μm on the wild‐type while enhancing its lifespan‐shortening effect on *clk‐1*. These observations suggested the possibility that the different compounds do not act on the same cell types or in the same cellular compartments or sites. To probe this notion further, we tested whether the effects of RSV and NAC on the wild‐type were additive. We maintained the concentration of RSV constant at 250 μm while increasing the concentration of NAC from 3 to 9 mm (Fig. 5A). The effects were additive at all concentrations of NAC. In particular, the effect of RSV was not less pronounced at the highest concentration of NAC than in the absence of NAC. In fact, the mix of the two compounds achieved a robust 50% increase in lifespan, substantially greater than those observed in any experiment with a single compound.

### Additive effects of RSV and PQ

The effects of PQ and RSV on *sod‐2;sod‐3* double mutants described above suggested that RSV might not act on mtROS levels. To test this further, we treated wild‐type worms simultaneously with a fixed concentration of RSV and varying concentration of PQ (Fig. 5B). Again, RSV and PQ appeared to act independently, with the effect of RSV being of identical magnitude at all concentrations of PQ, and with a large increase in lifespan (~45%) achieved at the highest concentration. As the action of RSV in *C. elegans* appears to be antioxidant in nature [as indicated by its strongly beneficial effect on *sod‐12345* quintuple mutants (Fig. 4C)], this additivity is difficult to explain unless PQ and RSV act in clearly different cell types or different sites within the same cells.

### Overall effects of antioxidants on oxidative stress

The compounds that we have tested are bona fide antioxidants as outlined in the introduction. However, they might also have other properties that could affect worm physiology. For example, NAC is a source of cysteine and might affect acid base as well as redox processes in addition to sulfur metabolism, VitC is an enzymatic cofactor, and many different properties have been claimed for RSV. Unfortunately, techniques to unequivocally determine cell‐specific or subcellular levels of ROS or oxidative damage are not currently available in *C. elegans*. Nonetheless, we sought to determine whether the antioxidant compounds we were testing were indeed capable of reducing overall ROS levels as assessed by DCF fluorescence, the most widely used test for oxidative stress (Fig. S2). We treated wild‐type worms with each of the three antioxidant compounds at the two concentrations for which clearly cut lifespan effects had been most readily observed. In all cases, DCF fluorescence was clearly decreased. However, in contrast to what was observed for lifespan, dose dependency was not observed. For example, RSV had essentially the same effects at both concentrations (50 and 250 μm), despite the fact that the effect on lifespan is substantially greater at 250 μm (Fig. 1a). Nor did the degree of reduction correlate with the magnitude of the effect on lifespan. For example, both VitC and RSV had a greater effect on DCF fluorescence than NAC, yet VitC has virtually no effect on lifespan of the wild‐type (Fig. 1C), and the effect of NAC at 9 mm is greater than that of RSV even at 250 μm (Fig. 1A,B). Taken together, we conclude from these observations that the DCF fluorescence test confirms the overall antioxidant properties of the tested compounds in our system, but does not provide a readout of the effect of the compounds on the processes that affect lifespan. The readout that was obtained might be due to large effects on major and easily accessible tissues such as the gut, which might not necessarily participate in the processes by which the antioxidants impact lifespan. The antioxidant effect on these tissues might however fully obscure more subtle effects on the cell types or the subcellular sites that are relevant for lifespan.

## Discussion

### ROS and aging

Until recently, ROS have been considered to be important determinants of the aging process because of their potential toxicity and because antioxidants were reputed to be beneficial. As reviewed in the introduction, these notions have recently been challenged by findings in model organisms that demonstrate beneficial effects on lifespan of increased ROS generation produced by mutations or pro‐oxidant treatments. For example, an inverted U‐shaped dose–response relationship was observed between the dosage of the pro‐oxidant PQ and lifespan (Yang & Hekimi, 2010a; Van Raamsdonk & Hekimi, 2012). Such a relationship would arise from a combination of beneficial effects from a moderate increase in ROS levels and their dose‐dependent toxicity.

As to the beneficial effects of a small increase in ROS levels, it has often been suggested that it could result from an increase in ROS defenses in response to ROS‐dependent damage, a mechanism sometimes called mitohormesis (Ristow, 2014; Yun & Finkel, 2014). Note that this notion already implies that ROS damage is at least in part the cause of aging. Another interpretation is that ROS are signaling molecules that are produced in response to molecular stresses resulting from the aging process but not particularly from oxidative stress. As signaling molecules, ROS then activate appropriate protective mechanisms. In this interpretation, the small elevation of ROS levels that increase lifespan is not stressful, nor do they induce an increased resistance to oxidative stress (Hekimi *et al*., 2011). We favor the latter interpretation because we have shown that much of ROS detoxification is irrelevant to normal lifespan (Van Raamsdonk & Hekimi, 2012), and because no gene expression‐dependent increase in detoxification accompanies lifespan‐enhancing pro‐oxidant treatment (Yee *et al*., 2014). Of course, a corollary of this interpretation is that we do not well understand the nature of the molecular stresses that accompany or cause aging.

In the present study, we used antioxidants rather than a pro‐oxidant to confirm the existence of an inverted U‐shaped dose–response relationship between ROS levels and lifespan, with both high and low levels of ROS appearing to be unfavorable for longevity. The fact that both antioxidant and pro‐oxidant treatments reveal such a relationship suggests that ROS levels are not optimized for lifespan in all cells, but are either too high or too low for maximum longevity in at least some cell types.

When an organism like *C. elegans* is treated with a compound, it is difficult to know which tissues and/or cell types are affected by the treatment and which are not: whether a sufficient concentration of the compound is obtained in a particular cell, or whether a given cell is actually sensitive to the compound. Similarly, it cannot easily be established in which subcellular compartment the compound is acting. However, the simple fact that a compound has an effect, and the conditions under which the compound has more or less of an effect, is informative. Past and present findings with PQ indicate that there are at least some cell types and subcellular compartments where increasing ROS is beneficial for lifespan (in the case of PQ, the compartment appears to be the mitochondria, but the target cell types are unknown). Similarly, the findings presented here, in particular the fact that antioxidants can increase longevity, indicate that there are cell types and compartments where ROS levels are too high for maximal longevity. On the other hand, our findings that enhancing the penetration of antioxidants with DMSO and *bus‐8* can lessen their pro‐longevity effects and even shorten lifespan indicate that in at least some cell types, ROS levels are relatively high, yet optimal for longevity.

### A model with multiple targets for pro‐oxidants and antioxidants

**Figure 6:**
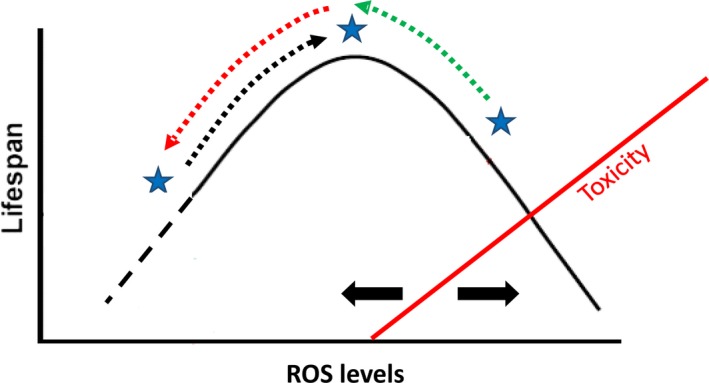
A model for the relationship between reactive oxygen species (ROS) levels and lifespan (see main text for discussion). 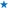, cell type‐, or subcellular site‐, specific ROS levels; 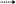, pro‐longevity effect of pro‐oxidants; 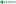, pro‐longevity effect of antioxidants; 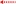, antilongevity effect of antioxidants; 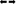, cell type‐specific differences in sensitivity to ROS toxicity.

We propose a model that can account for all of these observations (Fig. 6). The effect of any compound on the actual lifespan of an organism will result from the combined effects on all the cell types and compartments that the compound can affect. It will thus depend not only on the redox properties of the compound, but also on which cell types the compound can reach and affect (which is dependent on solvents used, ease of penetration into the organism, etc.). It is not known whether any single cell type displays by itself an inverted U‐shaped dose–response relationship between ROS levels and the longevity of the organism as a whole. However, the model does not require this. It remains valid even if the dose–response relationship results from the combination of the effect on several cell types. Similarly, any effect of a compound could result from the effects on several distinct subcellular sites of action rather than actions in different cells.

The effect of any compound on a particular mutant genotype will also depend on the particular pattern of changes to ROS levels produced by the genotype. These changes do not have to be uniform or unidirectional across all cells. Some mutant cells might have elevated and others normal or even lowered ROS levels. A good example of this is shown by the effect of PQ on *nuo‐6* mutants (Fig. 4A), where PQ is capable of further promoting the survival of mutants whose overall increased longevity is already due to elevated mtROS (Yang & Hekimi, 2010a). Thus, PQ likely acts on target cells that in these mutants still have ROS levels that are lower than what is most beneficial for longevity.

The model can also account for the observation of a U‐shaped (rather than inverted U‐shaped) dose–response relationship produced by the action of RSV on *gas‐1* and *nuo‐6* (Fig. 3D,E). Both these mutations affect complex I function and are known to alter ROS levels. At low concentration, RSV might succeed in reaching a cell type with a level of ROS that, in these mutants, is favorable for longevity, thus shortening lifespan. However, at higher concentration, RSV might reach additional cell types where ROS levels are higher than optimal for longevity, thus compensating for the lifespan‐shortening effect that is already displayed at low concentration, and accounting for the U‐shaped dose–response observed.

### Resveratrol and additivity

We observed that the effect of RSV was additive to the effects of both PQ and NAC (Fig. 5A,B). This observation supports our model as it suggests that the pro‐longevity effects of RSV on the wild‐type are due to effects at sites of action that are different from those that are targeted by PQ or NAC. However, as noted above, it is possible that two compounds act on the same cell type but in sufficiently different subcellular compartments to have additive effects. For example, although it is not known whether or not RSV acts on mitochondria in worms, our data of the absence of any alteration of the effect of RSV on *sod‐2;sod‐3* double mutants are consistent with this possibility. On the other hand, it seems clear that PQ acts in mitochondria (Yee *et al*., 2014). Thus, if RSV does not act in mitochondria but PQ mostly does, then any antioxidant effect of RSV might not be able to interfere with the superoxide‐generating effects of PQ. However, it is more difficult to see how the antioxidant effects of RSV and NAC could fail to affect the same processes if they acted in the same cells, given that NAC impinges on glutathione metabolism which in turn affects the redox state of the entire cell.

RSV has been proposed to act as an inhibitor or agonist of a number of specific molecular targets rather than as a redox‐active molecule (Bhullar & Hubbard, 2015; Bitterman & Chung, 2015). This could in principle explain the observed additivity with pro‐oxidant and antioxidant ROS modulators. However, this notion would need to be reconciled with the observations that RSV is highly sensitive to the altered redox conditions in mitochondrial mutants (Fig. 3) and that it acts like other antioxidants on mutants lacking superoxide detoxification (Fig. 4C). In any case, the results with RSV are not crucial to the conclusion that antioxidants can lengthen or shorten lifespan depending on concentration and the redox status of the target organism.

### Use of antioxidants in the health sciences

Our findings that ROS levels can be optimized for effects that are beneficial for survival suggest that antioxidants and pro‐oxidants can in principle be used positively to impact health. However, our findings also underline the need for compounds that affect the redox balance in well‐characterized ways. To ensure that the action of any antioxidant will in fact be beneficial in a particular pathological situation, it will be necessary to have very detailed information about the redox status of the tissues on which impact is sought as well as on how the treatment might impact any other tissue. Fortunately, these issues are now been addressed in considering antioxidant treatments for patients with cancer (Chandel & Tuveson, 2014).

## Experimental Procedures

### *C. elegans* strains and culture conditions

All strains were maintained by standard methods, at 20 °C, on NGM agar plates seeded with *Escherichia coli* OP50. The following mutations were used: N2 (wild‐type), *clk‐1(qm30), isp‐1(qm150), nuo‐6(qm200), gas‐1(fc21), bus‐8(e2698), sod‐1(tm783), sod‐2(ok1030), sod‐3(tm760), sod‐4(gk101),* and *sod‐5(tm1246)*.

### Lifespan analysis

Lifespan studies were performed at 20 °C. After hatching, worms were allowed to grow on plates containing the compound tested, or control plates. After reaching the L4 larval stage, worms were transferred onto new treatment plates containing 50 μm 5‐FUDR, which suppresses egg production. Worms were further transferred onto freshly prepared compound plates with FUDR once a week. Lost worms or worms dying from internal hatching despite FUDR were replaced from a backup pool.

### Resveratrol treatment

Resveratrol was dissolved in DMSO. RSV stock solution was spread onto NGM plates to a final concentration of 50 μm or 250 μm. Plates were seeded with OP50 once the RSV solution was absorbed into the agar.

### NAC and VitC treatments

*N*‐acetylcysteine (Sigma‐Aldrich, Oakville, Ontario, Canada) and VitC (Sigma‐Aldrich, Oakville, Ontario, Canada) were prepared in dH_2_O and added into NGM media to a final concentration of 3, 6, or 9 mm, and 5 and 10 mm, respectively. DMSO was spread on top of plates containing NAC or VitC, and control plates, to mimic the solvent conditions when using RSV. NAC alters *E. coli* OP50 growth. Therefore, NAC plates and control plates were spread with OP50 first grown on plates without NAC.

### Paraquat treatment

Paraquat (Sigma) was prepared in dH_2_O and added to NGM media to a final concentration of 0.1 mm or 0.0125 mm (in one experiment). Like NAC, PQ alters bacterial growth, and OP50 grown on separate plates without PQ were spread onto the plates with PQ as well as on control plates.

### Mixing treatments

For experiments mixing RSV and NAC or PQ, RSV was spread onto NAC or PQ plates to a final concentration of 250 μm. OP50 were grown on plates with 250 μm RSV and then smeared on experimental plates with NAC and RSV or NAC and PQ.

### Measurement of ROS levels

After exposure to the respective compounds from L1 larval stage on, worms were washed off of the plates with M9 buffer as young adults. Bacteria were removed by three repeated washes and subsequent centrifugation at low speed. Approximately 500 young adult worms in 50 μL M9 buffer were transferred in triplicate to the wells of 96‐well black microplates, followed by making up of the volume to 100 μL by addition of 2′,7′‐dichlorodihydrofluorescein diacetate (H_2_DCF‐DA; ThermoFisher Scientific, Waltham, MA USA), resulting in a final concentration of 50 μm. Fluorescence was read initially at the time of adding the dye and two hours after the addition of dye, using a microplate reader with 485 nm excitation and 520 nm emission. Initial readings were subtracted from the final readings and then normalized to protein content using the DC protein assay (Bio‐Rad, Mississauga, Ontario, Canada). The final results are expressed as percentage of relative DCF fluorescence with respect to the vehicle (DMSO)‐treated control within the same experimental preparation.

### Graphical representation and statistical analysis

All survival data are shown as percent change to the average lifespan compared to control. The *y*‐axis of most graphs shows these changes over a 60–140% range. When the range is different because of particularly large changes, this is indicated by arrows. In all cases, bars correspond to the pooled results of several independent trials each using > 50 worms. All numerical values from which the graphs were derived are given in Table S1 (Supporting information). To present survival data into percent change in mean lifespan compared to control, the binary raw lifespan information entered into Prism 5 for Windows (version 5.01; GraphPad) was converted to ‘day of death’ values using Microsoft Excel, where each ‘1′ value representing the death of an individual worm was transformed to its corresponding day of death value. Then, average lifespan was calculated. The average lifespan of the control was normalized to 100%, and the average lifespan of each condition was scaled accordingly. Standard error of the mean (SEM) was calculated using Microsoft Excel, and Student's *t*‐tests as well as log‐rank tests were performed.

## Author contributions

DD, BC, J‐LL, CY, KB, AK, and YW carried out the experiments. DD, LB, YW, and SH designed the experiments. SH wrote the manuscript.

## Conflict of Interest

None declared.

## Funding

No funding information provided.

## Supporting information


**Fig. S1** Survival curves corresponding to the experiments shown as bar graphs in the main text.

**Fig. S2** Reactive oxygen species (ROS) measurements following antioxidant treatments.

**Table. S1** Numerical values for all data plotted or described in the main text.

## References

[acel12528-bib-0001] Agarwal B , Baur JA (2011) Resveratrol and life extension. Ann. N. Y. Acad. Sci. 1215, 138–143. 21261652 10.1111/j.1749-6632.2010.05850.x

[acel12528-bib-0002] Ahmad KA , Clement MV , Pervaiz S (2003) Pro‐oxidant activity of low doses of resveratrol inhibits hydrogen peroxide‐induced apoptosis. Ann. N. Y. Acad. Sci. 1010, 365–373. 15033754 10.1196/annals.1299.067

[acel12528-bib-0003] Ahmad A , Syed FA , Singh S , Hadi SM (2005) Prooxidant activity of resveratrol in the presence of copper ions: mutagenicity in plasmid DNA. Toxicol. Lett. 159, 1–12. 15913925 10.1016/j.toxlet.2005.04.001

[acel12528-bib-0004] Bass TM , Weinkove D , Houthoofd K , Gems D , Partridge L (2007) Effects of resveratrol on lifespan in Drosophila melanogaster and *C. elegans* . Mech. Ageing Dev. 128, 546–552. 17875315 10.1016/j.mad.2007.07.007

[acel12528-bib-0005] Bhullar KS , Hubbard BP (2015) Lifespan and healthspan extension by resveratrol. Biochim. Biophys. Acta 1852, 1209–1218. 25640851 10.1016/j.bbadis.2015.01.012

[acel12528-bib-0006] Bitterman JL , Chung JH (2015) Metabolic effects of resveratrol: addressing the controversies. Cell. Mol. Life Sci. 72, 1473–1488. 25548801 10.1007/s00018-014-1808-8 PMC6324849

[acel12528-bib-0007] Chandel NS , Tuveson DA (2014) The promise and perils of antioxidants for cancer patients. N. Engl. J. Med. 371, 177–178. 25006725 10.1056/NEJMcibr1405701

[acel12528-bib-0008] Cocheme HM , Murphy MP (2008) Complex I is the major site of mitochondrial superoxide production by paraquat. J. Biol. Chem. 283, 1786–1798. 18039652 10.1074/jbc.M708597200

[acel12528-bib-0009] Ewbank JJ , Barnes TM , Lakowski B , Lussier M , Bussey H , Hekimi S (1997) Structural and functional conservation of the *C. elegans* timing gene clk‐1. Science 275, 980–983. 9020081 10.1126/science.275.5302.980

[acel12528-bib-0010] Felkai S , Ewbank JJ , Lemieux J , Labbe JC , Brown GG , Hekimi S (1999) CLK‐1 controls respiration, behavior and aging in the nematode *C. elegans* . EMBO J. 18, 1783–1792. 10202142 10.1093/emboj/18.7.1783 PMC1171264

[acel12528-bib-0011] Feng J , Bussiere F , Hekimi S (2001) Mitochondrial electron transport is a key determinant of life span in *C. elegans* . Dev. Cell 1, 633–644. 11709184 10.1016/s1534-5807(01)00071-5

[acel12528-bib-0012] Gadacha W , Ben‐Attia M , Bonnefont‐Rousselot D , Aouani E , Ghanem‐Boughanmi N , Touitou Y (2009) Resveratrol opposite effects on rat tissue lipoperoxidation: pro‐oxidant during day‐time and antioxidant at night. Redox Rep. 14, 154–158. 19695122 10.1179/135100009X466131

[acel12528-bib-0013] Galati G , Sabzevari O , Wilson JX , O'Brien PJ (2002) Prooxidant activity and cellular effects of the phenoxyl radicals of dietary flavonoids and other polyphenolics. Toxicology 177, 91–104. 12126798 10.1016/s0300-483x(02)00198-1

[acel12528-bib-0014] Gueguen N , Desquiret‐Dumas V , Leman G , Chupin S , Baron S , Nivet‐Antoine V , Vessieres E , Ayer A , Henrion D , Lenaers G , Reynier P , Procaccio V (2015) resveratrol directly binds to mitochondrial complex I and increases oxidative stress in brain mitochondria of aged mice. PLoS ONE 10, e0144290. 26684010 10.1371/journal.pone.0144290 PMC4694087

[acel12528-bib-0015] Hekimi S , Lapointe J , Wen Y (2011) Taking a “good” look at free radicals in the aging process. Trends Cell Biol. 21, 569–576. 21824781 10.1016/j.tcb.2011.06.008 PMC4074523

[acel12528-bib-0016] Holmstrom KM , Finkel T (2014) Cellular mechanisms and physiological consequences of redox‐dependent signalling. Nat. Rev. Mol. Cell Biol. 15, 411–421. 24854789 10.1038/nrm3801

[acel12528-bib-0017] Hwang AB , Ryu EA , Artan M , Chang HW , Kabir MH , Nam HJ , Lee D , Yang JS , Kim S , Mair WB , Lee C , Lee SS , Lee SJ (2014) Feedback regulation via AMPK and HIF‐1 mediates ROS‐dependent longevity in *C. elegans* . Proc. Natl Acad. Sci. USA 111, E4458–E4467. 25288734 10.1073/pnas.1411199111 PMC4210294

[acel12528-bib-0018] Kayser EB , Morgan PG , Hoppel CL , Sedensky MM (2001) Mitochondrial expression and function of GAS‐1 in *C. elegans* . J. Biol. Chem. 276, 20551–20558. 11278828 10.1074/jbc.M011066200

[acel12528-bib-0019] Labuschagne CF , Stigter EC , Hendriks MM , Berger R , Rokach J , Korswagen HC , Brenkman AB (2013) Quantification of in vivo oxidative damage in *C. elegans* during aging by endogenous F3‐isoprostane measurement. Aging Cell 12, 214–223. 23279719 10.1111/acel.12043

[acel12528-bib-0020] de la Lastra CA , Villegas I (2007) Resveratrol as an antioxidant and pro‐oxidant agent: mechanisms and clinical implications. Biochem. Soc. Trans. 35, 1156–1160. 17956300 10.1042/BST0351156

[acel12528-bib-0021] Miura T , Muraoka S , Ikeda N , Watanabe M , Fujimoto Y (2000) Antioxidative and prooxidative action of stilbene derivatives. Pharmacol. Toxicol. 86, 203–208. 10862501 10.1034/j.1600-0773.2000.d01-36.x

[acel12528-bib-0022] Morselli E , Maiuri MC , Markaki M , Megalou E , Pasparaki A , Palikaras K , Criollo A , Galluzzi L , Malik SA , Vitale I , Michaud M , Madeo F , Tavernarakis N , Kroemer G (2010) Caloric restriction and resveratrol promote longevity through the Sirtuin‐1‐dependent induction of autophagy. Cell Death Dis. 1, e10. 21364612 10.1038/cddis.2009.8 PMC3032517

[acel12528-bib-0023] Padayatty SJ , Katz A , Wang Y , Eck P , Kwon O , Lee JH , Chen S , Corpe C , Dutta A , Dutta SK , Levine M (2003) Vitamin C as an antioxidant: evaluation of its role in disease prevention. J. Am. Coll. Nutr. 22, 18–35. 12569111 10.1080/07315724.2003.10719272

[acel12528-bib-0024] Partridge FA , Tearle AW , Gravato‐Nobre MJ , Schafer WR , Hodgkin J (2008) The *C. elegans* glycosyltransferase BUS‐8 has two distinct and essential roles in epidermal morphogenesis. Dev. Biol. 317, 549–559. 18395708 10.1016/j.ydbio.2008.02.060

[acel12528-bib-0025] Queiroz AN , Gomes BA , Moraes WM Jr , Borges RS (2009) A theoretical antioxidant pharmacophore for resveratrol. Eur. J. Med. Chem. 44, 1644–1649. 18976835 10.1016/j.ejmech.2008.09.023

[acel12528-bib-0026] Ristow M (2014) Unraveling the truth about antioxidants: mitohormesis explains ROS‐induced health benefits. Nat. Med. 20, 709–711. 24999941 10.1038/nm.3624

[acel12528-bib-0027] Rushworth GF , Megson IL (2014) Existing and potential therapeutic uses for *N*‐acetylcysteine: the need for conversion to intracellular glutathione for antioxidant benefits. Pharmacol. Ther. 141, 150–159. 24080471 10.1016/j.pharmthera.2013.09.006

[acel12528-bib-0028] Schaar CE , Dues DJ , Spielbauer KK , Machiela E , Cooper JF , Senchuk M , Hekimi S , Van Raamsdonk JM (2015) Mitochondrial and cytoplasmic ROS have opposing effects on lifespan. PLoS Genet. 11, e1004972. 25671321 10.1371/journal.pgen.1004972 PMC4335496

[acel12528-bib-0029] Schulz TJ , Zarse K , Voigt A , Urban N , Birringer M , Ristow M (2007) Glucose restriction extends *C. elegans* life span by inducing mitochondrial respiration and increasing oxidative stress. Cell Metab. 6, 280–293. 17908557 10.1016/j.cmet.2007.08.011

[acel12528-bib-0030] Shibamura A , Ikeda T , Nishikawa Y (2009) A method for oral administration of hydrophilic substances to *C. elegans*: effects of oral supplementation with antioxidants on the nematode lifespan. Mech. Ageing Dev. 130, 652–655. 19580823 10.1016/j.mad.2009.06.008

[acel12528-bib-0031] Shibata Y , Branicky R , Landaverde IO , Hekimi S (2003) Redox regulation of germline and vulval development in *C. elegans* . Science 302, 1779–1782. 14657502 10.1126/science.1087167

[acel12528-bib-0032] Stivala LA , Savio M , Carafoli F , Perucca P , Bianchi L , Maga G , Forti L , Pagnoni UM , Albini A , Prosperi E , Vannini V (2001) Specific structural determinants are responsible for the antioxidant activity and the cell cycle effects of resveratrol. J. Biol. Chem. 276, 22586–22594. 11316812 10.1074/jbc.M101846200

[acel12528-bib-0033] Van Raamsdonk JM , Hekimi S (2009) Deletion of the mitochondrial superoxide dismutase sod‐2 extends lifespan in *C. elegans* . PLoS Genet. 5, e1000361. 19197346 10.1371/journal.pgen.1000361 PMC2628729

[acel12528-bib-0034] Van Raamsdonk JM , Hekimi S (2010) Reactive oxygen species and aging in *C. elegans*: causal or casual relationship? Antioxid. Redox Signal. 13, 1911–1953. 20568954 10.1089/ars.2010.3215

[acel12528-bib-0035] Van Raamsdonk JM , Hekimi S (2011) FUdR causes a twofold increase in the lifespan of the mitochondrial mutant gas‐1. Mech. Ageing Dev. 132, 519–521. 21893079 10.1016/j.mad.2011.08.006 PMC4074524

[acel12528-bib-0036] Van Raamsdonk JM , Hekimi S (2012) Superoxide dismutase is dispensable for normal animal lifespan. Proc. Natl Acad. Sci. USA 109, 5785–5790. 22451939 10.1073/pnas.1116158109 PMC3326508

[acel12528-bib-0037] Viswanathan M , Kim SK , Berdichevsky A , Guarente L (2005) A role for SIR‐2.1 regulation of ER stress response genes in determining *C. elegans* life span. Dev. Cell 9, 605–615. 16256736 10.1016/j.devcel.2005.09.017

[acel12528-bib-0038] Wood JG , Rogina B , Lavu S , Howitz K , Helfand SL , Tatar M , Sinclair D (2004) Sirtuin activators mimic caloric restriction and delay ageing in metazoans. Nature 430, 686–689. 15254550 10.1038/nature02789

[acel12528-bib-0039] Yang W , Hekimi S (2010a) A mitochondrial superoxide signal triggers increased longevity in *C. elegans* . PLoS Biol. 8, e1000556. 21151885 10.1371/journal.pbio.1000556 PMC2998438

[acel12528-bib-0040] Yang W , Hekimi S (2010b) Two modes of mitochondrial dysfunction lead independently to lifespan extension in *C. elegans* . Aging Cell 9, 433–447. 20346072 10.1111/j.1474-9726.2010.00571.x

[acel12528-bib-0041] Yang W , Li J , Hekimi S (2007) A measurable increase in oxidative damage due to reduction in superoxide detoxification fails to shorten the life span of long‐lived mitochondrial mutants of *C. elegans* . Genetics 177, 2063–2074. 18073424 10.1534/genetics.107.080788 PMC2219504

[acel12528-bib-0042] Yee C , Yang W , Hekimi S (2014) The intrinsic apoptosis pathway mediates the pro‐longevity response to mitochondrial ROS in *C. elegans* . Cell 157, 897–909. 24813612 10.1016/j.cell.2014.02.055 PMC4454526

[acel12528-bib-0043] Yun J , Finkel T (2014) Mitohormesis. Cell Metab. 19, 757–766. 24561260 10.1016/j.cmet.2014.01.011 PMC4016106

[acel12528-bib-0044] Zarse K , Schmeisser S , Birringer M , Falk E , Schmoll D , Ristow M (2010) Differential effects of resveratrol and SRT1720 on lifespan of adult *C. elegans* . Horm. Metab. Res. 42, 837–839. 20925017 10.1055/s-0030-1265225

[acel12528-bib-0045] Zheng LF , Wei QY , Cai YJ , Fang JG , Zhou B , Yang L , Liu ZL (2006) DNA damage induced by resveratrol and its synthetic analogues in the presence of Cu (II) ions: mechanism and structure‐activity relationship. Free Radic. Biol. Med. 41, 1807–1816. 17157183 10.1016/j.freeradbiomed.2006.09.007

