# Supplementary material for: Antioxidants reveal an inverted U‐shaped dose‐response relationship between reactive oxygen species levels and the rate of aging in Caenorhabditis elegans
Source: Aging Cell. 2016 Sep 28;16(1):104–12. doi: 10.1111/acel.12528 (PMC5242296; doi:10.1111/acel.12528)
Supplement: Supplementary file 1 — Fig. S1 Survival curves corresponding to the experiments shown as bar graphs in the main text. [file ACEL-16-104-s001.pdf]

FIGURE S1

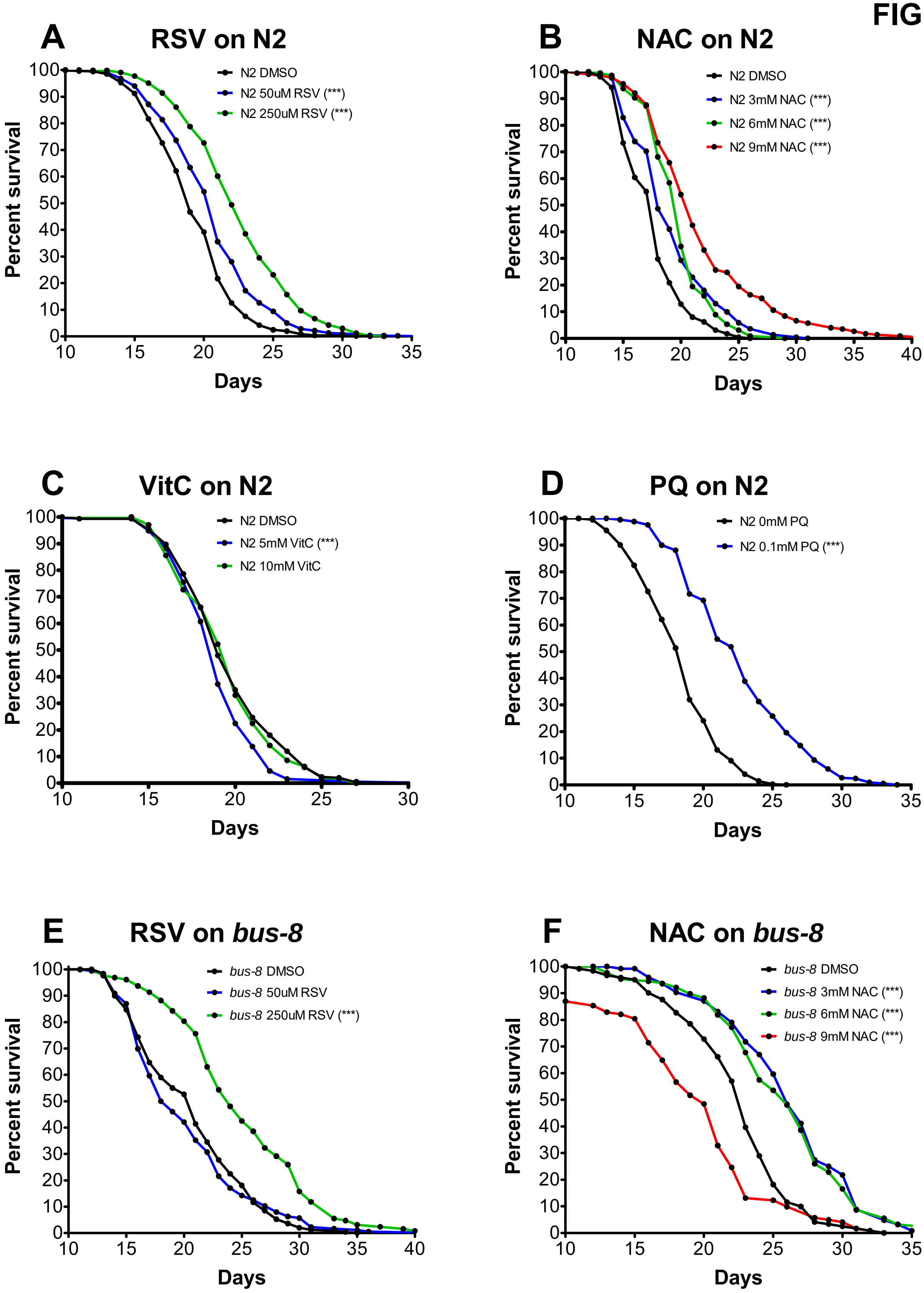
A
RSV on N2
N2 DMSO
N2 50uM RSV (***)
N2 250uM RSV (***)
Percent survival
Days
B
NAC on N2
N2 DMSO
N2 3mM NAC (***)
N2 6mM NAC (***)
N2 9mM NAC (***)
C
VitC on N2
N2 DMSO
N2 5mM VitC (***)
N2 10mM VitC
D
PQ on N2
N2 0mM PQ
N2 0.1mM PQ (***)
E
RSV on bus-8
bus-8 DMSO
bus-8 50uM RSV
bus-8 250uM RSV (***)
F
NAC on bus-8
bus-8 DMSO
bus-8 3mM NAC (***)
bus-8 6mM NAC (***)
bus-8 9mM NAC (***)

FIGURE S1

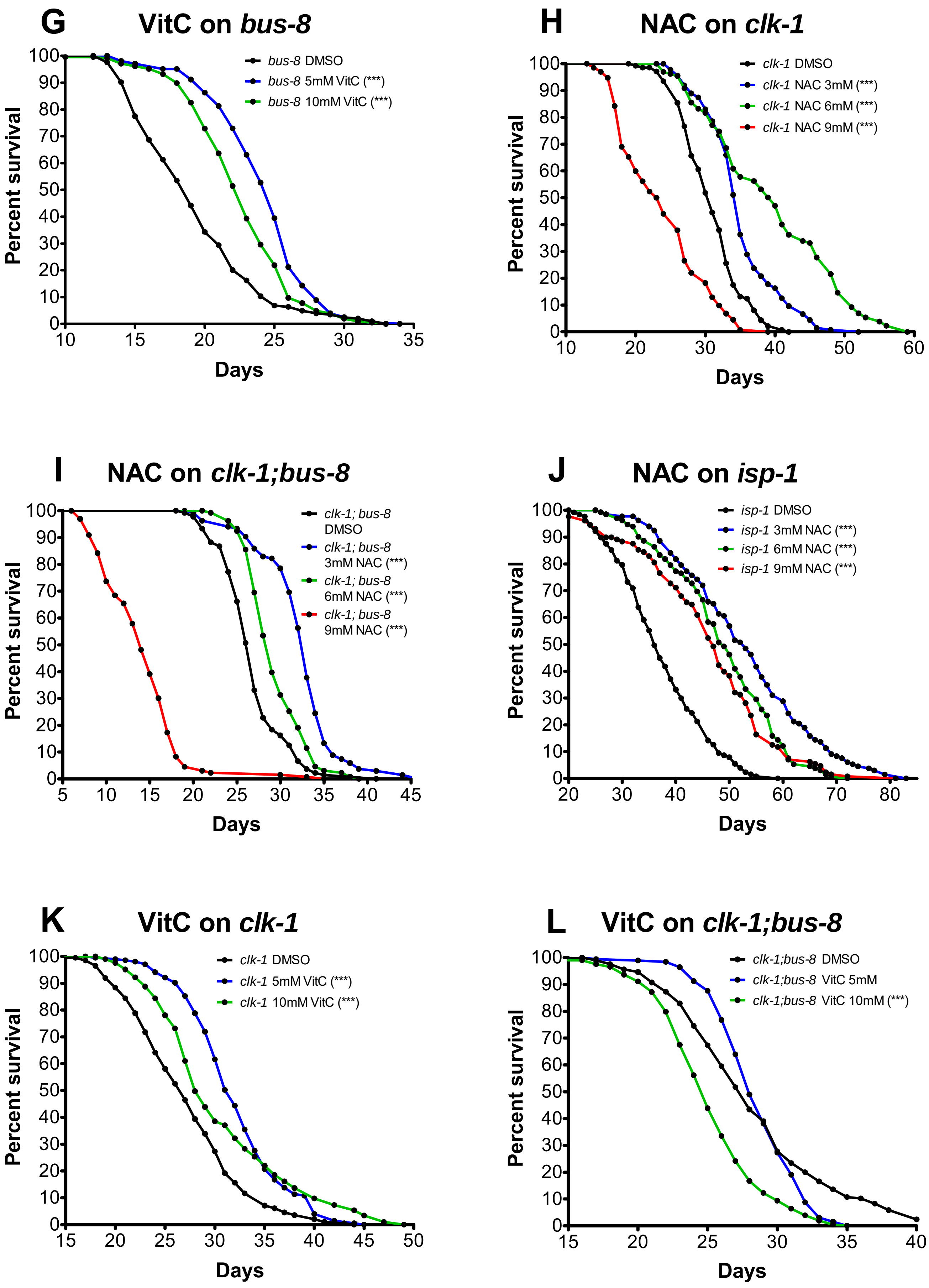
G
VitC on bus-8
bus-8 DMSO
bus-8 5mM VitC (***)
bus-8 10mM VitC (***)
Percent survival
Days
H
NAC on clk-1
clk-1 DMSO
clk-1 NAC 3mM (***)
clk-1 NAC 6mM (***)
clk-1 NAC 9mM (***)
Percent survival
Days
I
NAC on clk-1;bus-8
clk-1; bus-8 DMSO
clk-1; bus-8 3mM NAC (***)
clk-1; bus-8 6mM NAC (***)
clk-1; bus-8 9mM NAC (***)
Percent survival
Days
J
NAC on isp-1
isp-1 DMSO
isp-1 3mM NAC (***)
isp-1 6mM NAC (***)
isp-1 9mM NAC (***)
Percent survival
Days
K
VitC on clk-1
clk-1 DMSO
clk-1 5mM VitC (***)
clk-1 10mM VitC (***)
Percent survival
Days
L
VitC on clk-1;bus-8
clk-1;bus-8 DMSO
clk-1;bus-8 VitC 5mM
clk-1;bus-8 VitC 10mM (***)
Percent survival
Days

FIGURE S1

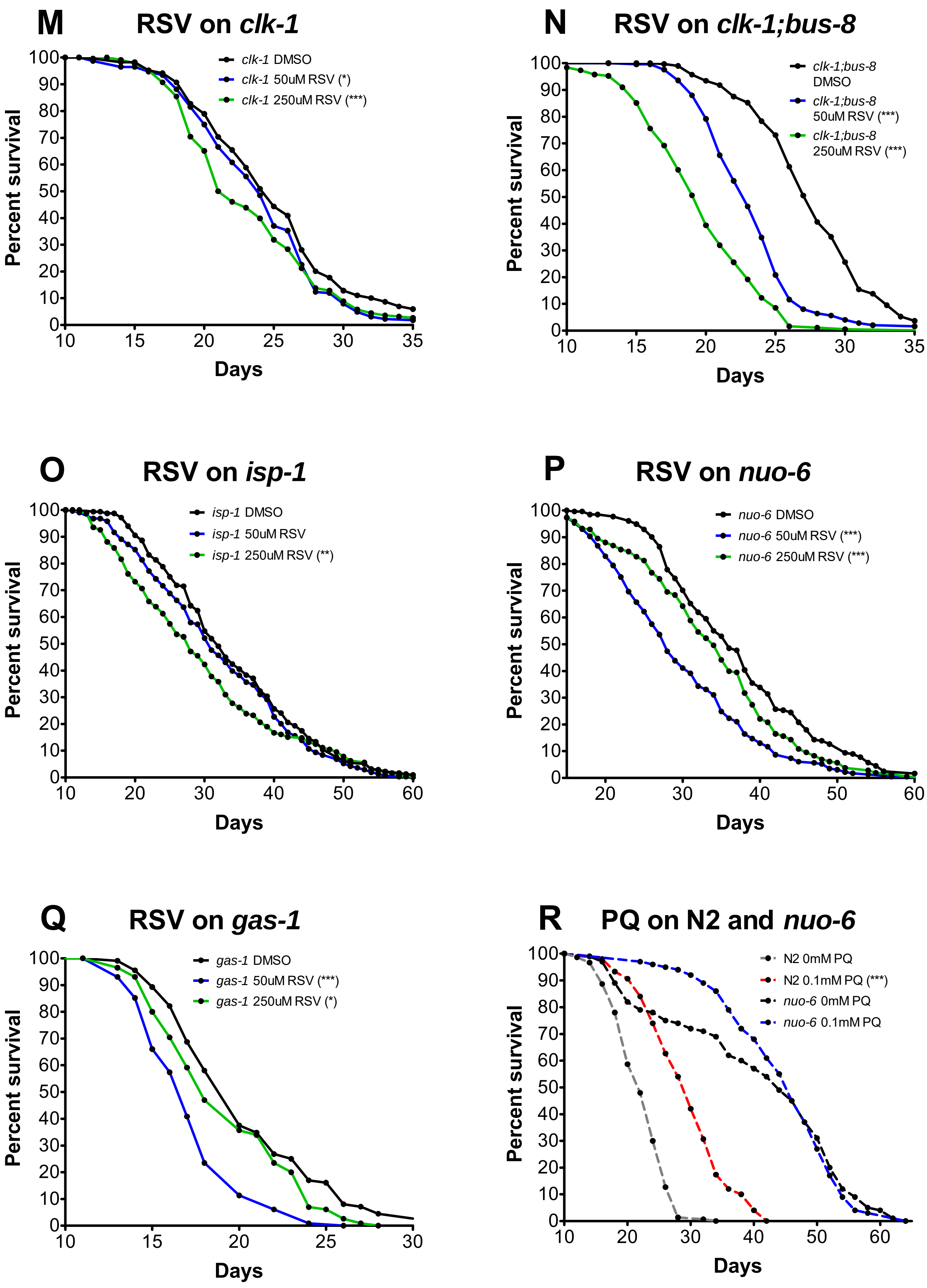
M
RSV on clk-1
clk-1 DMSO
clk-1 50uM RSV (*)
clk-1 250uM RSV (***)
Percent survival
Days
N
RSV on clk-1;bus-8
clk-1;bus-8 DMSO
clk-1;bus-8 50uM RSV (***)
clk-1;bus-8 250uM RSV (***)
O
RSV on isp-1
isp-1 DMSO
isp-1 50uM RSV
isp-1 250uM RSV (**)
P
RSV on nuo-6
nuo-6 DMSO
nuo-6 50uM RSV (***)
nuo-6 250uM RSV (***)
Q
RSV on gas-1
gas-1 DMSO
gas-1 50uM RSV (***)
gas-1 250uM RSV (*)
R
PQ on N2 and nuo-6
N2 0mM PQ
N2 0.1mM PQ (***)
nuo-6 0mM PQ
nuo-6 0.1mM PQ

FIGURE S1

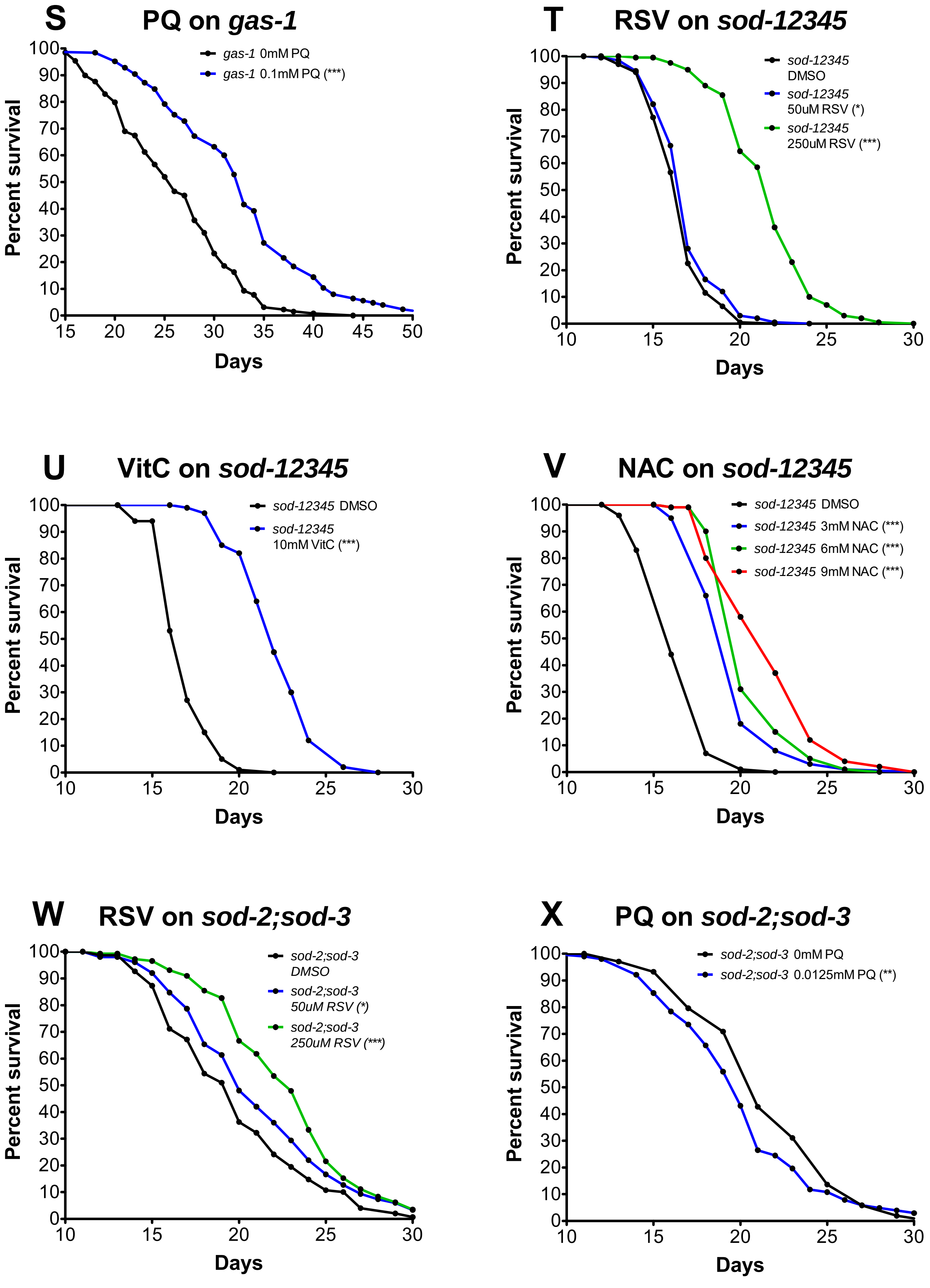
S
PQ on gas-1
gas-1 0mM PQ
gas-1 0.1mM PQ (***)
Percent survival
Days
T
RSV on sod-12345
sod-12345 DMSO
sod-12345 50uM RSV (*)
sod-12345 250uM RSV (***)
Percent survival
Days
U
VitC on sod-12345
sod-12345 DMSO
sod-12345 10mM VitC (***)
Percent survival
Days
V
NAC on sod-12345
sod-12345 DMSO
sod-12345 3mM NAC (***)
sod-12345 6mM NAC (***)
sod-12345 9mM NAC (***)
Percent survival
Days
W
RSV on sod-2;sod-3
sod-2;sod-3 DMSO
sod-2;sod-3 50uM RSV (*)
sod-2;sod-3 250uM RSV (***)
Percent survival
Days
X
PQ on sod-2;sod-3
sod-2;sod-3 0mM PQ
sod-2;sod-3 0.0125mM PQ (**)
Percent survival
Days

FIGURE S1

Y NAC and RSV on N2

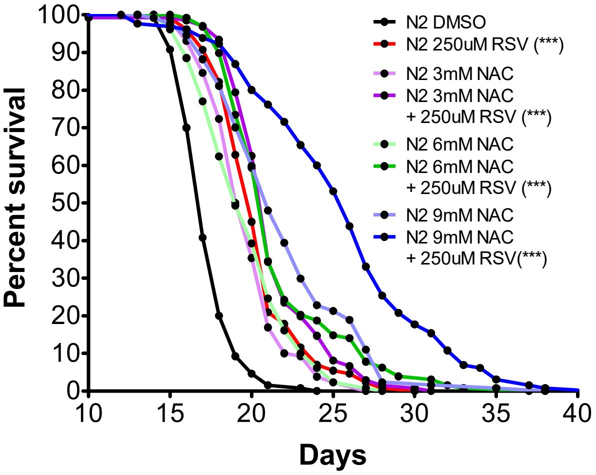

Z PQ and RSV on N2-1

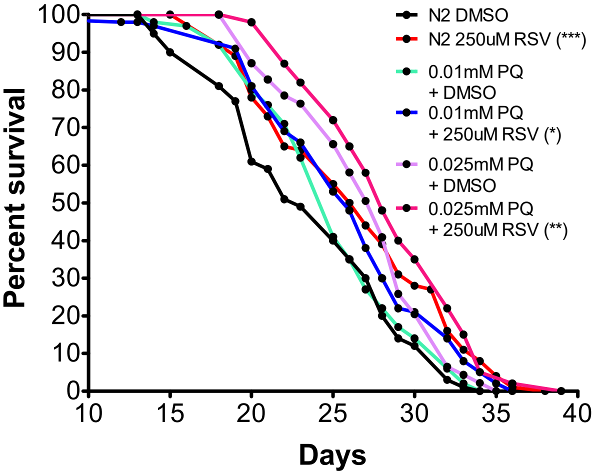

AA PQ and RSV on N2-2

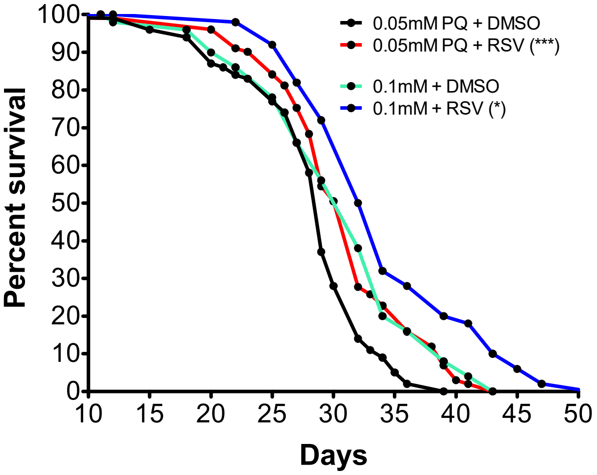

Legend

Figure S1 Corresponding lifespan survival plots for lifespan data presented in the form of bar graphs within the article. The following survival plots are ordered according to the appearance of their corresponding bar graphs. **A)** The effect of RSV on the lifespan of N2. **B)** The effect of NAC on the lifespan of N2. **C)** The effect of VitC on the lifespan of N2. **D)** The effect of PQ on the lifespan of N2. **E)** The effect of RSV on the lifespan of *bus-8*. **F)** The effect of NAC on the lifespan of *bus-8*. **G)** The effect of VitC on the lifespan of *bus-8*. **H)** The effect of NAC on the lifespan of *clk-1*. **I)** The effect of NAC on the lifespan of *clk-1;bus-8*. **J)** The effect of NAC on the lifespan of *isp-1*. **K)** The effect of VitC on the lifespan of *clk-1*. **L)** The effect of VitC on the lifespan of *clk-1;bus-8*. **M)** The effect of RSV on the lifespan of *clk-1*. **N)** The effect of RSV on the lifespan of *clk-1;bus-8*. **O)** The effect of RSV on the lifespan of *isp-1*. **P)** The effect of RSV on the lifespan of *nuo-6*. **Q)** The effect of RSV on the lifespan of *gas-1*. **R)** The effect of PQ on the lifespan of N2 and *nuo-6*. **S)** The effect of PQ on the lifespan of *gas-1*. **T)** The effect of RSV on the lifespan of *sod-12345*. **U)** The effect of VitC on the lifespan of *sod-12345*. **V)** The effect of NAC on the lifespan of *sod-12345*. **W)** The effect of RSV on the lifespan of *sod-2;sod-3*. **X)** The effect of PQ on the lifespan of *sod-2;sod-3*. **Y)** The effect of NAC and RSV on the lifespan of N2. **Z)** The effect of PQ and RSV on the lifespan and N2 (part 1 of 2). **AA)** The effect of PQ and RSV on the lifespan of N2 (part 2 of 2). Please refer to the main article for additional details. Survival curves show the percentage of worms alive as a function of time. The y-axes show percent survival from 0 to 100%. The x-axes show the age of the worms, and may vary between plots, since they are individualized to separate survival lines for different conditions as much as possible. The asterisks indicate P-values from log-rank comparisons to the control: * represents 0.01 to 0.05; ** = represents 0.001 to 0.01; *** represents <0.001.
