# Supplementary material for: Antioxidants reveal an inverted U‐shaped dose‐response relationship between reactive oxygen species levels and the rate of aging in Caenorhabditis elegans
Source: Aging Cell. 2016 Sep 28;16(1):104–12. doi: 10.1111/acel.12528 (PMC5242296; doi:10.1111/acel.12528)
Supplement: Supplementary file 2 — Fig. S2 Reactive oxygen species (ROS) measurements following antioxidant treatments. [file ACEL-16-104-s002.pdf]

Figure S2

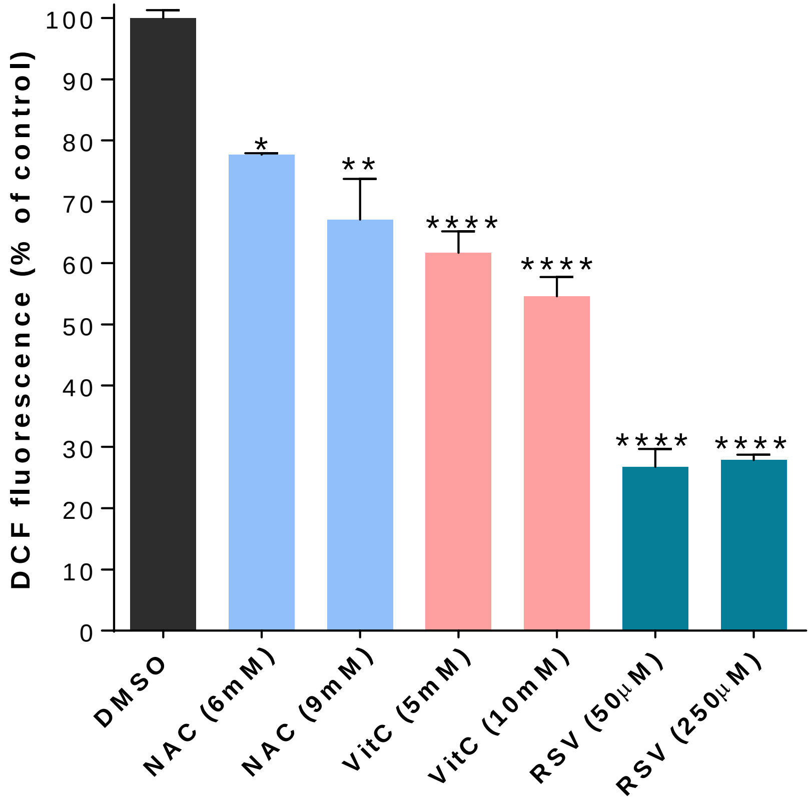

Effect of different treatments on ROS levels. Relative ROS levels in wild-type N2 worms after different treatments were measured using a the H2DCF-DA assay. NAC, N-Acetyl-Cysteine; VitC, vitamin C; RSV, resveratrol. Data are means $\pm$ SEM (n = 3); *p<0.05, **p<0.01, ****p<0.0001 relative to DMSO-treated controls (one-way ANOVA followed by post hoc Dunnett's test).
