# Supplementary material for: Antioxidants reveal an inverted U‐shaped dose‐response relationship between reactive oxygen species levels and the rate of aging in Caenorhabditis elegans
Source: Aging Cell. 2016 Sep 28;16(1):104–12. doi: 10.1111/acel.12528 (PMC5242296; doi:10.1111/acel.12528)
Supplement: Supplementary file 3 — Table. S1 Numerical values for all data plotted or described in the main text. [file ACEL-16-104-s003.pdf]

TABLE S1

Genotype / treatment	Number of repeats	Sample size (N)	Mean lifespan in days ± SEM	Maximum lifespan in days	P-value compared to control (t-test)	P-value compared to control (log rank test)
Wild-type / DMSO	18	N= 973	19.39 ± 0.09665	32		
Wild-type / 50µM RSV	17	N= 978	20.66 ± 0.1131	38	P< 0.0001	P< 0.0001
Wild-type / 250µM RSV	18	N= 1034	22.62 ± 0.1172	33	P< 0.0001	P< 0.0001
Wild-type / DMSO	4	N= 225	17.64 ± 0.1734	25		
Wild-type / 3mM NAC	4	N= 222	19.24 ± 0.2387	30	P< 0.0001	P< 0.0001
Wild-type / 6mM NAC	4	N= 226	19.85 ± 0.1754	28	P< 0.0001	P< 0.0001
Wild-type / 9mM NAC	4	N= 226	21.96 ± 0.3455	41	P< 0.0001	P< 0.0001
Wild-type / DMSO	5	N= 300	19.76 ± 0.1655	31		
Wild-type / 5mM VitC	3	N= 196	19.05 ± 0.1670	33	P= 0.0039	P= 0.0004
Wild-type / 10mM VitC	5	N= 303	19.61 ± 0.1556	27	ns	ns
Wild-type	8	N= 419	18.37 ± 0.1400	26		
Wild-type / 0.1mM PQ	8	N= 419	22.73 ± 0.1971	34	P< 0.0001	P< 0.0001
Wild-type / 9mM NAC (- DMSO)	2	N= 128	18.06 ± 0.1993	23		
Wild-type / 9mM NAC (+ DMSO)	2	N= 127	19.95 ± 0.2190	26	P< 0.0001	P< 0.0001
bus-8 (e2698) / DMSO	4	N= 249	20.57 ± 0.3028	35		
bus-8 (e2698) / 50µM RSV	4	N= 176	20.17 ± 0.3915	42	ns	ns
bus-8 (e2698) / 250µM RSV	4	N= 127	25.02 ± 0.5126	43	P< 0.0001	P< 0.0001
bus-8 (e2698) / DMSO	2	N= 121	22.40 ± 0.3692	33		
bus-8 (e2698) / 3mM NAC	2	N= 124	26.06 ± 0.4277	36	P< 0.0001	P< 0.0001
bus-8 (e2698) / 6mM NAC	2	N= 127	25.71 ± 0.4584	41	P< 0.0001	P< 0.0001
bus-8 (e2698) / 9mM NAC	2	N= 122	18.86 ± 0.5766	33	P< 0.0001	P= 0.0003
bus-8 (e2698) / DMSO	3	N= 204	19.34 ± 0.2995	33		
bus-8 (e2698) / 5mM VitC	3	N= 203	24.22 ± 0.2436	34	P< 0.0001	P< 0.0001
bus-8 (e2698) / 10mM VitC	3	N= 206	22.60 ± 0.2540	33	P< 0.0001	P< 0.0001
clk-1 (qm30) / DMSO	2	N= 137	30.89 ± 0.3667	42		
clk-1 (qm30) / 3mM NAC	2	N= 135	35.06 ± 0.4479	52	P< 0.0001	P< 0.0001
clk-1 (qm30) / 6mM NAC	2	N= 130	39.55 ± 0.8035	59	P< 0.0001	P< 0.0001
clk-1 (qm30) / 9mM NAC	2	N= 132	23.78 ± 0.5244	39	P< 0.0001	P< 0.0001
clk-1 (qm30); bus-8 (e2698) / DMSO	2	N= 136	26.82 ± 0.3084	41		
clk-1 (qm30); bus-8 (e2698) / 3mM NAC	2	N= 135	32.39 ± 0.3795	46	P< 0.0001	P< 0.0001
clk-1 (qm30); bus-8 (e2698) / 6mM NAC	2	N= 131	29.36 ± 0.2759	39	P< 0.0001	P< 0.0001
clk-1 (qm30); bus-8 (e2698) / 9mM NAC	2	N= 133	17.86 ± 0.6307	35	P< 0.0001	P< 0.0001
isp-1 (qm150) / DMSO	2	N= 127	37.25 ± 0.7328	59		
isp-1 (qm150) / 3mM NAC	2	N= 132	53.02 ± 1.067	83	P< 0.0001	P< 0.0001
isp-1 (qm150) / 6mM NAC	2	N= 132	48.81 ± 0.8773	72	P< 0.0001	P< 0.0001
isp-1 (qm150) / 9mM NAC	2	N= 128	46.29 ± 1.065	81	P< 0.0001	P< 0.0001
clk-1 (qm30) / DMSO	3	N= 198	27.19 ± 0.3973	44		
clk-1 (qm30) / 5mM VitC	3	N= 203	32.08 ± 0.3387	45	P< 0.0001	P< 0.0001
clk-1 (qm30) / 10mM VitC	3	N= 205	30.57 ± 0.4721	49	P< 0.0001	P< 0.0001
clk-1 (qm30); bus-8 (e2698) / DMSO	3	N= 205	28.34 ± 0.3902	45		
clk-1 (qm30); bus-8 (e2698) / 5mM VitC	3	N= 194	28.58 ± 0.2123	35	ns	ns
clk-1 (qm30); bus-8 (e2698) / 10mM VitC	3	N= 203	25.17 ± 0.2679	35	P< 0.0001	P< 0.0001
clk-1 (qm30) / DMSO	4	N= 289	25.10 ± 0.3421	49		
clk-1 (qm30) / 50µM RSV	4	N= 227	24.03 ± 0.3299	42	P= 0.0275	P= 0.0259
clk-1 (qm30) / 250µM RSV	4	N= 226	23.29 ± 0.3510	44	P< 0.0001	P= 0.0009
clk-1 (e2519) / DMSO	6	N= 325	26.52 ± 0.2802	48		
clk-1 (e2519) / 50µM RSV	6	N= 329	25.41 ± 0.2770	44	P= 0.0049	P= 0.0126
clk-1 (e2519) / 250µM RSV	6	N= 330	22.96 ± 0.2923	42	P< 0.0001	P< 0.0001
clk-1 (qm30); bus-8 (e2698) / DMSO	5	N= 305	27.73 ± 0.2513	42		
clk-1 (qm30); bus-8 (e2698) / 50µM RSV	4	N= 250	23.32 ± 0.2356	43	P< 0.0001	P< 0.0001
clk-1 (qm30); bus-8 (e2698) / 250µM RSV	3	N= 188	19.62 ± 0.3019	36	P< 0.0001	P< 0.0001
isp-1 (qm150) / DMSO	6	N= 316	33.78 ± 0.5878	65		
isp-1 (qm150) / 50µM RSV	6	N= 310	32.06 ± 0.6060	60	P= 0.0434	ns (P= 0.0773)
isp-1 (qm150) / 250µM RSV	6	N= 309	29.60 ± 0.6661	64	P< 0.0001	P= 0.0011
nuo-6 (qm200) / DMSO	5	N= 246	37.18 ± 0.6195	65		
nuo-6 (qm200) / 50µM RSV	5	N= 249	29.33 ± 0.5940	65	P< 0.0001	P< 0.0001
nuo-6 (qm200) / 250µM RSV	5	N= 253	33.31 ± 0.6151	62	P< 0.0001	P= 0.0009
gas-1 (fc21) / DMSO	2	N= 112	20.40 ± 0.4161	33		
gas-1 (fc21) / 50µM RSV	2	N= 115	17.26 ± 0.2720	26	P < 0.0001	P < 0.0001
gas-1 (fc21) / 250µM RSV	2	N= 115	19.23 ± 0.3550	28	P= 0.0323	P= 0.0237
isp-1 (qm150); bus-8 (e2698) / DMSO	3	N= 194	30.98 ± 0.6813	60		
isp-1 (qm150); bus-8 (e2698) / 50µM RSV	2	N= 132	32.45 ± 0.7793	55	ns	ns
isp-1 (qm150); bus-8 (e2698) / 250µM RSV	2	N= 136	28.43 ± 0.6512	48	P= 0.0093	P= 0.0009
gas-1 (kn1)	2	N= 129	25.88 ± 0.5326	44		
gas-1 (kn1) / 0.1mM PQ	2	N= 125	32.53 ± 0.6764	54	P < 0.0001	P < 0.0001
sod-12345 / DMSO	4	N= 200	16.66 ± 0.1120	22		
sod-12345 / 50µM RSV	4	N= 200	17.04 ± 0.1265	24	P= 0.0282	P= 0.0168
sod-12345 / 250µM RSV	4	N= 200	21.71 ± 0.1797	30	P < 0.0001	P < 0.0001
sod-12345 / DMSO	2	N= 100	16.90 ± 0.1446	22		
sod-12345 / 10mM VitC	2	N= 100	22.30 ± 0.2259	28	P < 0.0001	P < 0.0001
sod-12345 / DMSO	2	N= 100	16.66 ± 0.1810	22		
sod-12345 / 3mM NAC	2	N= 100	19.84 ± 0.2214	30	P < 0.0001	P < 0.0001
sod-12345 / 6mM NAC	2	N= 100	20.82 ± 0.2052	28	P < 0.0001	P < 0.0001
sod-12345 / 9mM NAC	2	N= 100	21.84 ± 0.2936	30	P < 0.0001	P < 0.0001
sod-2 (ok1030) ; sod-3 (tm760) / DMSO	3	N= 149	19.80 ± 0.3439	32		
sod-2 (ok1030) ; sod-3 (tm760) / 50µM RSV	3	N= 150	21.11 ± 0.3716	34	P= 0.0103	P= 0.0138
sod-2 (ok1030) ; sod-3 (tm760) / 250µM RSV	3	N= 144	22.83 ± 0.3526	38	P < 0.0001	P < 0.0001
sod-2 (ok1030) ; sod-3 (tm760)	2	N= 103	21.72 ± 0.3930	31		
sod-2 (ok1030) ; sod-3 (tm760) / 0.1mM PQ	2	N= 102	20.12 ± 0.4259	34	P= 0.0062	P= 0.0071
Wild-type / DMSO	2	N= 130	17.38 ± 0.1438	24		
Wild-type / 3mM NAC	2	N= 130	19.78 ± 0.2081	27		
Wild-type / 6mM NAC	2	N= 130	19.75 ± 0.2450	28		
Wild-type / 9mM NAC	2	N= 127	21.98 ± 0.3630	38		
Wild-type / 250µM RSV	2	N= 129	20.47 ± 0.2293	30	P < 0.0001	P < 0.0001
Wild-type / 3mM NAC + 250µM RSV	2	N= 136	21.40 ± 0.2392	31	P < 0.0001	P < 0.0001
Wild-type / 6mM NAC + 250µM RSV	2	N= 128	21.75 ± 0.3196	37	P < 0.0001	P < 0.0001
Wild-type / 9mM NAC + 250µM RSV	2	N= 130	25.58 ± 0.4747	41	P < 0.0001	P < 0.0001
Wild-type / DMSO	2	N= 100	23.59 ± 0.5426	34		
Wild-type / 0.01mM PQ	2	N= 100	25.03 ± 0.4563	34		
Wild-type / 0.025mM PQ	2	N= 99	26.46 ± 0.4570	35		
Wild-type / 0.05mM PQ	2	N= 100	28.09 ± 0.5211	39		
Wild-type / 0.1mM PQ	2	N= 50	30.62 ± 0.9458	43		
Wild-type / 250µM RSV	2	N= 100	26.40 ± 0.5580	38	P < 0.0001	P= 0.0002
Wild-type / 0.01mM PQ + 250µM RSV	2	N= 100	25.97 ± 0.5254	36	ns	P= 0.0403
Wild-type / 0.025mM + 250µM RSV	2	N= 100	28.53 ± 0.4439	39	P= 0.0014	P= 0.0056
Wild-type / 0.05mM + 250µM RSV	2	N= 101	30.76 ± 0.5592	43	P < 0.0001	P= 0.0001
Wild-type / 0.1mM + 250µM RSV	2	N= 50	34.22 ± 0.9365	51	P= 0.0081	P= 0.0122
